# Predictive accuracy of left atrial strain parameters for risk of atrial fibrillation recurrence: a systematic review and meta-analysis

**DOI:** 10.3389/fmed.2026.1798092

**Published:** 2026-04-20

**Authors:** Liping Wu, Youfu He, Lei Peng, Li Liu, Xiulong Tao

**Affiliations:** 1 Department of Geriatrics, Linping Hospital of Integrated Traditional Chinese and Western Medicine, Hangzhou, Zhejiang, China; 2 Department of Cardiology, Guizhou Provincial People's Hospital, Guiyang, Guizhou, China; 3 Department of Cardiology, Linping Hospital of Integrated Traditional Chinese and Western Medicine, Hangzhou, Zhejiang, China; 4 Department of Cardiology, Jinan Integrated Traditional Chinese and Western Medicine Hospital, Jinan, Shandong, China; 5 Department of Cardiology, Wuxi No.2 Chinese Medicine Hospital, Wuxi, Jiangsu, China

**Keywords:** atrial fibrillation recurrence, left atrial strain parameter, meta-analysis, prediction, systematic review

## Abstract

**Background:**

Clinical evidence, in recent years, has increased regarding the application of left atrial strain parameters for the prediction of atrial fibrillation (AF) recurrence. This study endeavors to assess the predictive power of these parameters for AF recurrence.

**Methods:**

We systematically searched Cochrane Library, Embase, PubMed, and Web of Science from inception to October 20, 2025, for cohort studies investigating the association between AF recurrence and various left atrial strain parameters, including peak atrial longitudinal strain (PALS, P-wave-triggered), left atrial reservoir strain (LASr, R-wave-triggered), left atrial conduit strain (LAScd), and left atrial contraction strain (LASct). A random-effects model was used to pool risk ratios (RRs) and predictive performance metrics. Sensitivity analysis, publication bias assessment, and subgroup analysis were performed.

**Results:**

Totally 25 studies covering 3,649 patients were included. The meta-analysis indicated that PALS and LASr measured before treatment were effective predictors for AF recurrence. Analyzed as categorical variables, both a higher PALS (RR = 0.08, 95% CI: 0.04–0.16) and a higher LASr (RR = 0.91, 95% CI: 0.86–0.96) were linked to a significantly lower risk of AF relapse. Treated as continuous variables, a 1-unit increase in PALS (RR = 0.88, 95% CI: 0.85–0.91) or LASr (RR = 0.93, 95% CI: 0.88–0.99) was associated with a pronounced lower risk of AF recurrence. The pooled AUC values for PALS and LASr were 0.75 and 0.78, respectively. The predictive power of other parameters was limited or unclear: LASct and LAScd measured before treatment, as well as LASr measured after treatment (either as a categorical variable or a continuous variable), failed to show significant predictive power (all *P* > 0.05). Only for LASct measured after treatment as a continuous variable, each unit elevation in LASct was linked to a reduced risk of AF relapse (RR = 0.75, 95% CI: 0.63–0.91).

**Conclusion:**

This study suggests that lower PALS and LASr values are associated with a higher risk of AF recurrence. In addition, PALS and LASr shows relatively favorable predictive performance for AF recurrence.

**Systematic review registration:**

https://www.crd.york.ac.uk/PROSPERO/view/CRD420251182805, identifier: CRD420251182805.

## 1 Introduction

Atrial fibrillation (AF) is one of the most prevalent sustained arrhythmias in clinical settings. The incidence rate and prevalence rate of AF climb annually due to population aging and the increasing prevalence of cardiovascular risk factors (1). AF significantly elevates the risk of heart failure, ischemic stroke, or all-cause mortality, severely lowers patients' quality of life, and imposes a heavy burden on social medical care (2). Although rhythm control strategies, including drug therapy, radiofrequency catheter ablation, and surgical intervention, have become important approaches for maintaining sinus rhythm, alleviating symptoms, and ameliorating prognosis, AF recurrence remains highly prevalent (3, 4). Previous studies indicate that the recurrence rate of AF following an initial catheter ablation procedure is approximately 20%−45%, and many need to undergo ablation again (5, 6). The early identification of high-risk populations for AF recurrence, both before and after treatment, represents a key challenge in current clinical practice.

Left atrial myopathy is pivotal to the onset and perpetuation of AF (7). Enduring pressure and volume overload lead to left atrial dilation, increased wall stress, and interstitial fibrosis, resulting in dual remodeling based on anatomy and electrophysiology, thereby providing a substrate for the triggers and maintenance of AF (8). Consequently, assessing the structure and function of the left atrium has long been regarded as an important approach to predicting AF recurrence (7, 9). In recent years, echocardiography based on speckle-tracking has served as a novel tool for the non-invasive evaluation of the function of the left atrium (10). By tracking the displacement of speckles within the atrial myocardium throughout the cardiac cycle, longitudinal strain parameters of the left atrium at different functional stages can be obtained. These parameters include peak atrial longitudinal strain (PALS, measured with P-wave triggering), left atrial reservoir strain (LASr, measured with R-wave triggering), left atrial conduit strain (LAScd), and left atrial contraction strain (LASct) (11). Although PALS and LASr both reflect left atrial reservoir function, they are not strictly synonymous due to their reference to different ECG timing points. These strain parameters are not reliant on geometric assumptions and are more sensitive to segmental and early functional alterations, holding the potential to earlier and more comprehensively reveal the reconstruction of the left atrium and the abnormality of left atrium—left ventricle coupling compared with conventional structural indicators.

Currently, multiple cohort studies have demonstrated that reduced left atrial strain parameters, including PALS and LASr, are closely associated with AF recurrence following catheter ablation or electrical cardioversion, suggesting the potential prognostic value of left atrial strain parameters (12–14). Nevertheless, findings regarding LASct and LAScd are inconsistent, and no consensus has been reached on their predictive performance for the risk of AF recurrence (15, 16). To date, there is a lack of a comprehensive, quantitative evaluation and comparison of the predictive power of different left atrial strain parameters for AF recurrence through a systematic review and meta-analysis. Therefore, this study endeavors to comprehensively explore the associations between left atrial strain parameters (PALS, LASct, LASr, and LAScd) and AF recurrence, and to evaluate the predictive performance of these parameters for AF relapse. In this study, PALS and LASr were analyzed as separate parameters, as both reflect left atrial reservoir function but are derived from different ECG triggering points, namely P-wave triggering for PALS and R-wave triggering for LASr. The goal is to provide a higher level of evidence for the clinical application of left atrial strain parameters in risk stratification of individuals with AF recurrence, and to offer a reference to developing individualized rhythm control strategies and follow-up management.

## 2 Materials and methods

### 2.1 Protocol registration

The protocol for this meta-analysis was registered on the International Prospective Register of Systematic Reviews (PROSPERO) (Registration number: CRD420251182805). No deviations from the registered protocol occurred in this study. This meta-analysis was implemented adhering to the Preferred Reporting Items for Systematic Reviews and Meta-Analyses (PRISMA) guidelines (17).

### 2.2 Literature search

Four databases, comprising PubMed, Web of Science, Embase, and Cochrane Library, were thoroughly searched from the commencement of each database to October 20, 2025. The key terms identified for the search included peak atrial longitudinal strain or PALS, left atrial contraction strain, or LASct, left atrial reservoir strain or LASr, left atrial conduit strain or LAScd, and atrial fibrillation or AF. Medical Subject Headings (MeSH) and free-text terms were combined using Boolean operators (AND, OR, and NOT). The search strategy is detailed in Supplementary Table 1. Furthermore, a backward citation tracking was conducted for systematic reviews, narrative reviews, and all included studies. Due to the lack of full standardization of terminology in the literature, both PALS and LASr were included in the search strategy and were screened according to the ECG reference point reported in the original studies.

### 2.3 Inclusion and exclusion criteria and study selection

The inclusion criteria comprised: (i) studies enrolled participants with a documented history of paroxysmal or persistent AF; (ii) studies utilized a cohort design; (iii) studies reported left atrial strain parameters covering PALS, LASr, LASct, and/or LAScd; (iv) extracted data included risk ratio (RR) alongside its 95% confidence interval (CI), specificity, sensitivity, and area under the summary receiver operating characteristic (SROC) curve (AUC) for the association of these strain parameters with AF relapse; (v) studies involved patients with AF who had undergone interventions for rhythm control, including catheter ablation (radiofrequency or cryopreservation balloon), surgical ablation, or electrical cardioversion. In this study, PALS was defined as left atrial reservoir strain derived from P-wave triggering, and LASr as that derived from R-wave triggering. Accordingly, these two parameters were extracted and analyzed separately rather than being treated as interchangeable. Studies were excluded provided that they were: (i) letters, reviews, case reports, conference abstracts, or commentaries; (ii) animal studies; (iii) studies with overlapping or duplicate data; (iv) studies from which the full text was unavailable; (v) were not published in English.

The titles and abstracts of the records initially identified were screened independently by two investigators (LW and XT). The full texts of the potentially eligible studies were further reviewed. All disagreements were reconciled by consensus with a third investigator (YH).

### 2.4 Data extraction

Two researchers (LP and LL) independently assessed the eligible studies and extracted relevant data. The following data were collected: country, publication year, first author's name, study population, study design, sample size, sex, AF type, body mass index (BMI), procedure type, heart failure, coronary artery disease, diabetes mellitus, hypertension, left ventricular ejection fraction (LVEF), left atrial strain parameters, follow-up duration, and timing of measurement. If data on true positive, false positive, false negative, or true negative were not directly provided in the literature, they would be calculated by the two investigators based on reported data on sensitivity and specificity. Any discrepancy between the two investigators was reconciled through consultation with a third researcher (LW).

### 2.5 Quality assessment

The quality of studies included in the diagnostic analysis was assessed utilizing the Quality Assessment of Diagnostic Accuracy Studies-−2 (QUADAS-2) tool in RevMan 5.3 (18). The risk of bias for each included study was evaluated, and the applicability of each included study in four key domains was assessed, comprising patient selection, index test, reference standard, and flow and timing. For studies in which only a correlation analysis was performed, the quality was appraised utilizing the Newcastle-Ottawa scale (NOS) (19). The NOS assesses 3 aspects, consisting of comparability (2 points), selection (4 points), and the adequacy of outcome and follow-up (3 points), with a total score varying between 0 and 9. A study with an NOS score >6 was categorized as high-quality. Two researchers (XT and YH) independently evaluated the risk of bias for each study included, and all disagreements were reconciled by consensus.

### 2.6 Statistical analysis

Each statistical analysis was implemented utilizing Stata 15.0 and Meta-Disc 1.4. Initially, heterogeneity among the included studies was examined. The degree of heterogeneity was determined based on the *I*^2^ statistic and the associated *P*-value. *I*^2^ ≤ 50% and *P* > 0.1 signified low heterogeneity, and a fixed-effects model was adopted for the meta-analysis; otherwise, a random-effects model was selected. RRs alongside their 95% CIs were pooled, and subgroup analysis and meta-regression were performed to probe into possible sources of heterogeneity. Sensitivity analysis was carried out by sequentially eliminating an individual study to assess the robustness of the pooled results. Publication bias was evaluated leveraging Egger's test and through funnel plots. For diagnostic data, the presence of a threshold effect was tested in Meta-DiSc 1.4 to compute the Spearman's correlation coefficient between the logit of sensitivity and the logit of (1–specificity) and its corresponding *P*-value. A *P*-value >0.05 was considered an absence of threshold effect. Based on the heterogeneity assessment, an appropriate effect model was employed for the meta-analysis to calculate pooled specificity, sensitivity, negative likelihood ratio (NLR), positive likelihood ratio (PLR), diagnostic odds ratio (DOR), and AUC. An AUC between 0.5 and 0.7 indicates low predictive value, an AUC ranging from 0.7 to 0.9 reflects moderate predictive performance, and an AUC of 0.9 or higher suggests high predictive accuracy. All statistical tests were two-sided, and statistical significance was defined as *P* < 0.05.

## 3 Results

### 3.1 Characteristics of included studies

**Figure 1:**
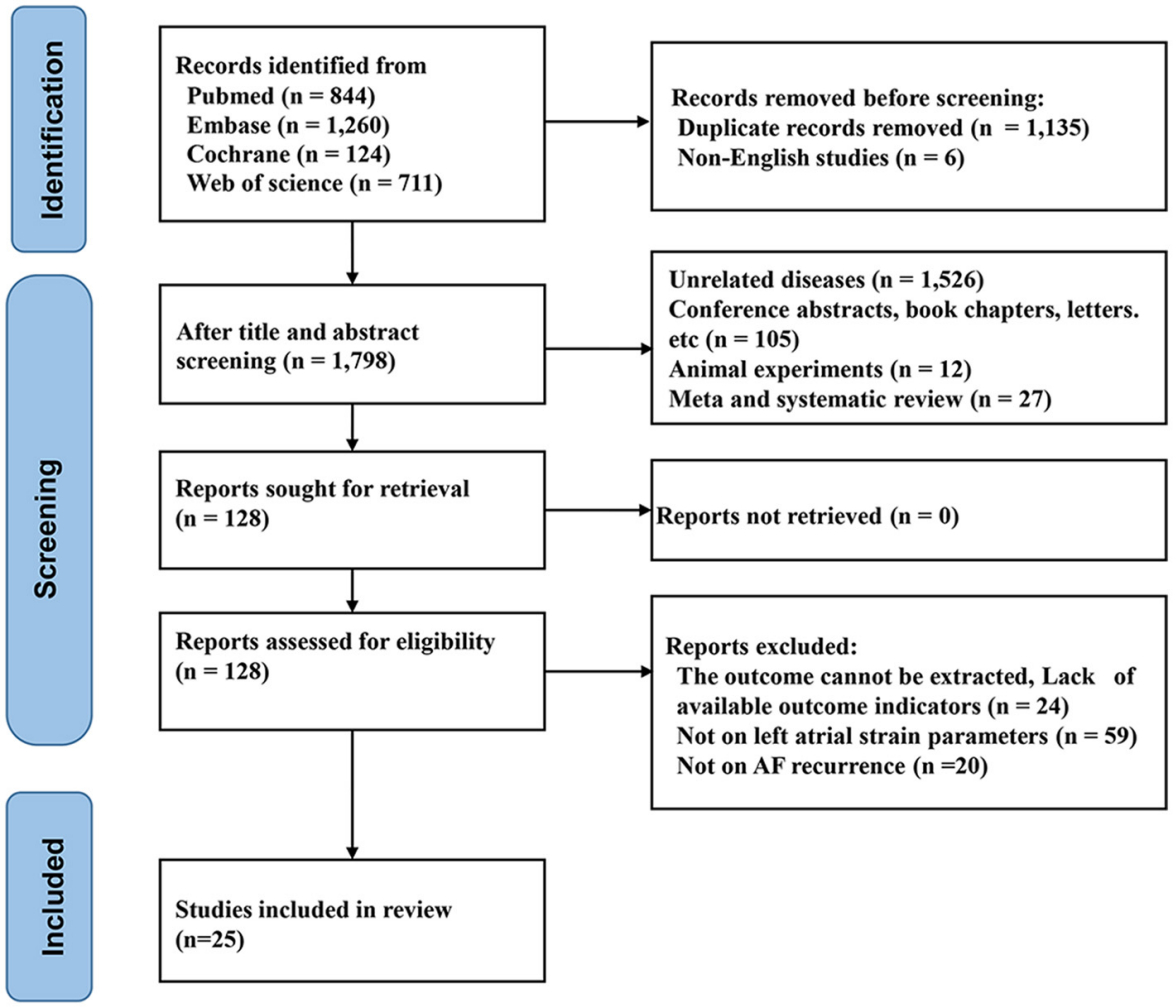
Flow diagram for study selection.

Totally 2,939 records were identified from the four databases. Among these, 1,135 duplicate records, 6 non-English publications, and 12 animal studies were eliminated. Following a review of the titles and abstracts of the remaining records, 1,658 studies were removed. The full texts of the remaining 128 studies were then assessed. Of these, 103 studies were excluded. Ultimately, 25 studies (10, 12, 14–16, 20–39) were included (Figure 1). The number of included patients varied between 42 and 678. The cumulative sample size was 3,649 patients. The average age was 61.62 ± 10.35 years. The detailed characteristics of the included studies are presented in Table 1. The mean + standard deviation of left atrial strain parameters measured both before and after treatment for AF recurrence is shown in Supplementary Table 2.

**Table 1:** Characteristics of included studies.

Author	Year	Indicators	Country	Study design	Sample size	Age	Male (%)	BMI	Types of atrial fibrillation	Treatment	Hyper-tension	Heart failure	Diabetes	LVEF (%)	Coronary artery disease	Measurement time	Follow-up period	Diagnosis of recurrence	Measuring technique	Software
Anastasio	2025	LASr, LASct, LAScd	Italy	Prospective cohort study	61	66.4 ± 9.1	83.6	27.0 ± 5.4	Persistent atrial fibrillation	Electrical conversion	80.3	18	NA	NA	9.8	post-treatment	6 months	ECG, Holter, electronic patient record	2D-STE	EchoPAC (GE)
Antonio Moreno-Ruiz	2019	PALS	Mexico	Prospective cohort study	131	67.23 ± 12.07	58.02	28.56 ± 3.47	PnVAF: 38.16%; LSPnVAF: 61.84%	Electrical conversion	87.79	0	25.95	66.66 ± 6.15	0	pre-treatment	6 months	ECG, Holter	2D-STE	QLAB (Philips)
Baalman	2022	LASr, LASct, LAScd	Netherlands	Retrospective cohort study	204	58.6 ± 7.8	73	27.5 ± 3.9	Persistent atrial fibrillation (57%), paroxysmal atrial fibrillation (43%)	Surgical ablation	42	4	6	NA	NA	pre-treatment	12 months	ECG, Holter	CMR feature-based tracking	CVI42 (Circle)
Bai	2018	PALS	China	Retrospective cohort study	87	61.91 ± 10.59	71.26	25.90 ± 3.73	Paroxysmal atrial fibrillation (74.71%) and persistent atrial fibrillation (25.29%)	RF ablation	57.47	NA	24.14	61.84 ± 4.58	17.24	pre-treatment	10.3 ± 6.4 months	Holter	2D-STE	NA
Benjamin	2022	LASr, LASct, LAScd	America	Retrospective cohort study	80	58.6 ± 9.4	75	NA	Paroxysmal atrial fibrillation	Catheter ablation	47.5	10	3.8	60 ± 9	12.5	pre-treatment + post-treatment	48 months	ECG, Holter, event recorder	CMR tissue tracking	CVI42
Bras	2024	LASr, LASct, LAScd	Portugal	Retrospective cohort study	78	59 ± 11	65	28.2 ± 4.2	Paroxysmal atrial fibrillation (69%), persistent atrial fibrillation (17%), long-standing persistent atrial fibrillation (14%)	RF ablation	69	NA	10	53.7 ± 10.2	NA	pre-treatment	12 months	ECG, Holter, external loop recorder	2D-STE	EchoPAC (GE)
Cho	2023	LASr	Korea	Prospective cohort study	61	49.5 ± 8.1	78.7	24.8 ± 2.8	Paroxysmal atrial fibrillation	RF ablation	24.6	0	8.2	56.3 ± 4.4	0	pre-treatment	12 months	Holter, event recorder	2D-STE	EchoPAC (GE)
Gastl	2022	LASr, LAScd	Germany	Retrospective cohort study	52	66.8 ± 10.1	53.8	26.3 ± 4.3	Paroxysmal atrial fibrillation (69.2%), persistent atrial fibrillation (30.8%)	Cryoballoon ablation/ RF ablation	48.1	1.9	1.9	63.9 ± 8.2	28.8	post-treatment	12 months	ECG, Holter	CMR feature-based tracking	CVI42
Hanaki	2020	LASr	Japan	Retrospective cohort study	100	63 ± 9	84	24.8 ± 3.4	Long-standing persistent atrial fibrillation	RF ablation	54	NA	11	62.3 ± 9.2	13	pre-treatment	34 ± 16 months	ECG, Holter, portable electrocar-diogram recorder	2D-STE	EchoPAC (GE)
Hao	2024	LASr	China	Prospective cohort study	80	62.46 ± 8.45	66.2	25.54 ± 5.49	Paroxysmal atrial fibrillation (70%), persistent atrial fibrillation (30%)	RF ablation	55	0	10	60.44 ± 8.47	NA	pre-treatment	12 months	ECG	2D-STE + 3D-STE	EchoPAC
Hong	2013	LASr, LAScd	China	Prospective cohort study	45	51.78 ± 6.83	67	25.21 ± 2.53	Paroxysmal solitary atrial fibrillation	RF ablation	0	0	0	68.07 ± 6.26	0	pre-treatment	3 months	ECG, Holter	2D-STE	EchoPAC (GE)
Hopman	2023	LASr, LASct, LAScd	Netherlands	Retrospective cohort study	110	60 ± 10	66	26.2 ± 3.7	Paroxysmal atrial fibrillation (72%), non-paroxysmal atrial fibrillation (28%)	RF ablation	34	7	5	NA	4	pre-treatment	12 months	ECG, Holter	CMR feature-based tracking	CVI42
Khan	2023	LASr, LASct, LAScd	England	Prospective cohort study	83	63.6 ± 9.7	73.5	30.5 ± 5.0	Long-standing persistent atrial fibrillation	Catheter ablation/ surgical ablation	51.8	NA	7.2	56.1 ± 7.5	13.3	pre-treatment + post-treatment	12 months	Implantable circulation recorder	2D-STE	TomTec
Li	2022	LASr, LASct, LAScd	China	Retrospective cohort study	95	63.2 ± 9.7	56.8	25.8 ± 3.3	Paroxysmal atrial fibrillation (70.5%), persistent atrial fibrillation (29.5%)	RF ablation	63.2	NA	18.9	57.64 ± 6.78	45.5	pre-treatment	25.79 ± 1.50 months	ECG, Holter	2D-STE	EchoPAC (GE)
Kiliszek	2023	LASr, LAScd	Poland	Retrospective cohort study	199	62.5 ± 10.6	61.3	30.1 ± 4.8	Paroxysmal atrial fibrillation (62.3%), persistent atrial fibrillation (37.7%)	RF ablation	72.9	25.6	20.6	60.3 ± 5.97	NA	pre-treatment	12 ± 5.23 months	ECG, Holter	2D-STE	EchoPAC (GE)
Marchandise	2022	PALS	Belgium	Prospective cohort study	94	65 ± 9	73	29 ± 5	Persistent atrial fibrillation	RF ablation	53	NA	12	56 ± 3.9	14	pre-treatment	8.65 ± 3.76 months	ECG, Holter, symptom-driven ECG and event recorder	2D-STE	Image-Arena (TomTec)
Mochizuki	2017	PALS	Belgium	Prospective cohort study	42	58 ± 10	69	25 ± 3	Paroxysmal atrial fibrillation	RF ablation	43	NA	7	66 ± 7	NA	pre-treatment	14.7 months	ECG, Holter	3D-STE	Toshiba Artida
Motoc	2021	PALS, PACS	Belgium	Prospective cohort study	172	62.6 ± 12.2	61	27.4 ± 4.8	Paroxysmal atrial fibrillation (83.7%), persistent atrial fibrillation (16.3%)	Cryoballoon ablation	59.9	8.1	10.5	54.5 ± 18.1	19.8	pre-treatment	11.7 ± 1.6 months	ECG, Holter	2D-STE	EchoPAC (GE)
Nielsen	2022	LASr, LASct, LAScd	Denmark	Retrospective cohort study	678	62.71 ± 10.4	72	NA	Paroxysmal atrial fibrillation (72%), persistent atrial fibrillation (24%), long-standing persistent atrial fibrillation (4%)	Cryoballoon ablation/ RF ablation	40	8	6	60.8 ± 6.24	7	pre-treatment	12 ± 0.89 months	ECG, Holter, event recorder	2D-STE	EchoPAC (GE)
Song	2025	PALS, LASr, LASct, LAScd	China	Prospective cohort study	195	62.6 ± 11.95	67.69	24.91 ± 3.52	Paroxysmal atrial fibrillation:32%, persistent atrial fibrillation:68%	RF ablation	43	12	13	59.41 ± 9.40	18	pre-treatment	11.0 ± 2.8 months	ECG, Holter	2D-STE	EchoPAC (GE)
Soysal	2024	LASr, LASct, LAScd	Turkey	Prospective cohort study	92	59 ± 11.7	54.3	28.8 ± 4.6	Paroxysmal atrial fibrillation (79.3%), persistent atrial fibrillation (20.7%)	Cryoballoon ablation	54.3	NA	25	NA	10.9	pre-treatment + post-treatment	24 months	Holter	2D-STE	Qlab (Philips)
Walek	2020	LASr, LASct, LAScd	Poland	Prospective cohort study	89	64.13 ± 9.35	66.29	31.17 ± 4.95	Persistent atrial fibrillation	Electrical conversion	86.52	NA	20.22	60.78 ± 9.72	13.48	post-treatment	12 months	ECG, Holter	2D-STE	EchoPAC (GE)
Zeng	2024	LASr, LAScd	China	Prospective cohort study	380	59.4 ± 10.8	72.1	NA	Paroxysmal atrial fibrillation (60.5%), persistent atrial fibrillation (39.5%)	Catheter ablation	50	6.6	14.2	63.14 ± 10.91	19.2	pre-treatment	10.05 ± 9.67 months	ECG, Holter	2D-STE	NA
Zhang	2025	LASr, LASct, LAScd	China	Prospective cohort study	109	62.4 ± 8.7	59.6	22.5 ± 2.3	Paroxysmal atrial fibrillation	RF Ablation	47.7	0	14.7	60.5 ± 5.0	12.8	pre-treatment + post-treatment	12.2 months	ECG, Holter	3D-STE	EchoPAC (GE)
Zheng	2023	PALS	China	Retrospective cohort study	332	61.39 ± 6.37	64.5	23.41 ± 3.20	Paroxysmal atrial fibrillation (68.1%), persistent atrial fibrillation (31.9%)	RF ablation	48.2	NA	15.9	57.39 ± 4.82	NA	pre-treatment	3~12 months	ECG, Holter	2D-STE	QLAB (Philips)

AF, atrial fibrillation; LASr, reservoir strain; LASct, contraction strain; LAScd, left atrial conduit strain; PALS, peak atrial longitudinal strain; BMI, body mass index; LVEF, left ventricular ejection fraction; PnVAF, persistent non-valvular AF; LSPnVAF, long standing persistent non-valvular AF; 2D-STE, two-dimensional speckle tracking echocardiography; 3D-STE, three-dimensional speckle tracking echocardiography; ECG, Electrocardiogram. RF, Radiofrequency.

### 3.2 Quality of included studies

**Figure 2:**
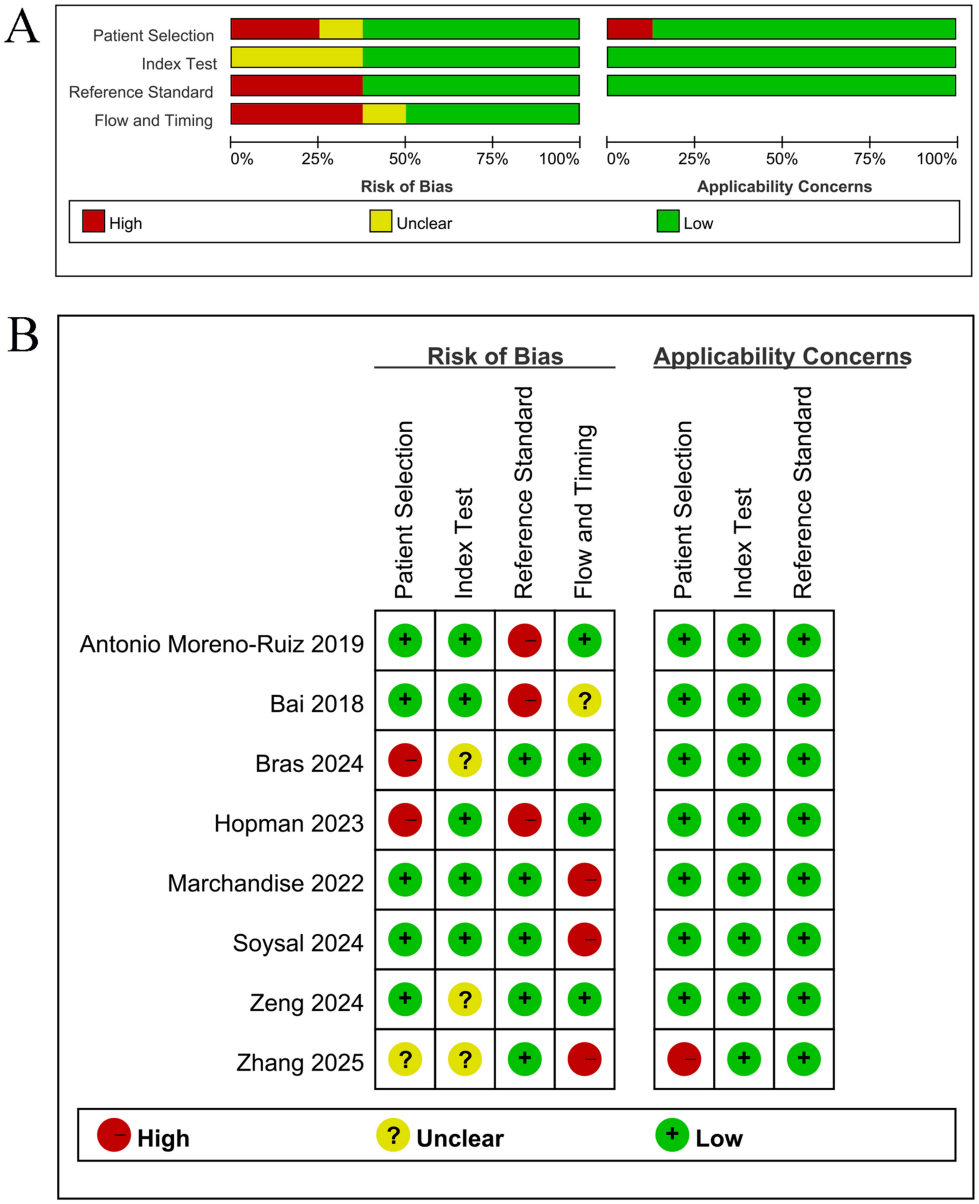
**(A)** Bar chart for risk of bias item and applicability, **(B)** Summary chart for risk of bias item and applicability.

Quality assessment utilizing the QUADAS-2 tool is illustrated in Figures 2A, B. Overall, some heterogeneity was observed in the methodological quality of the included studies. Specifically, in the domain of patient selection, two studies (16, 28) were identified as having a high risk of bias, and one study (12) was assessed to have an unclear risk, indicating potential selection bias. In the domain of index test, two studies (16, 39) were rated to have an unclear risk of bias, primarily owing to insufficient reporting regarding the blinding procedures. In the domain of reference standard, two studies (10, 28) were judged to have a high risk of bias, and one (22) was rated as having an unclear risk. Notably, concerns were more prominent in the domain of flow and timing, where three studies (12, 32, 37) were assessed to have a high risk of bias, and one (22) was judged to have an unclear risk. The concerns in the domain of flow and timing possibly impacted the estimates of diagnostic accuracy significantly.

The NOS scores for the included studies varied between 6 and 9, signifying high quality. The detailed NOS scores are provided in Supplementary Table 3.

### 3.3 Meta-analysis results

#### 3.3.1 PALS and AF recurrence

**Figure 3:**
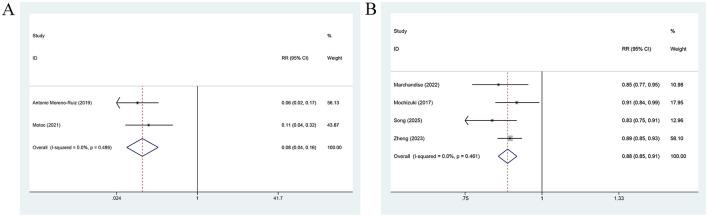
Forest plot for the association between AF recurrence and PALS measured before treatment, analyzed as a categorical variable **(A)**, and a continuous variable **(B)**.

PALS measured before treatment was significantly associated with AF recurrence prior to treatment. When analyzed as a categorical variable [2 studies (10, 34); Figure 3A], a higher PALS was linked to a significantly lower risk of AF recurrence in comparison with a lower PALS (RR = 0.08, 95% CI: 0.04–0.16, *P* = 0.001). When treated as a continuous variable [4 studies (14, 32, 33, 36); Figure 3B], each unit increase in PALS was associated with an approximately 12% lower risk of AF recurrence (RR = 0.88, 95% CI: 0.85–0.91, *P* = 0.001). No heterogeneity was observed in either analysis (*I*^2^ = 0%), and a fixed-effects model was applied. The subgroup analysis further demonstrated that PALS was consistently and significantly effective in predicting the risk of AF relapse across different regions, AF types, and study designs (Table 2).

**Table 2:** Subgroup analysis of association between left atrial strain parameters and AF recurrence.

Subgroup	PALS (continuous variable)			
	Study	RR [95% CI]	*P* value	*I* ^2^
**Total**	4	0.88 [0.85–0.91]	0.001	0%,
Study design				
Retrospective cohort study	1	0.89 [0.85–0.93]	0.001	NA
Prospective cohort study	3	0.87 [0.82–0.92]	0.001	11%
Population				
paroxysmal AF	1	0.91[0.84–0.99]	0.024	NA
Persistent AF	1	0.85 [0.77–0.94]	0.002	NA
Mixed AF	2	0.88 [0.84–0.91]	0.001	32.50%
Region				
Europe	2	0.89 [0.83–0.95]	0.001	0.50%
Asia	2	0.88 [0.84–0.91]	0.001	32.50%
Subgroup	LASr (dichotomous variables)			
	Study	RR [95% CI]	*P* value	*I* ^2^
**Total**	7	0.91 [0.86–0.96]	0.052	74.20%
Study design				
Retrospective cohort study	6	0.89 [0.81–0.97]	0.008	78.40%
Prospective cohort study	1	0.93 [0.90–0.96]	0.001	NA
Population				
paroxysmal AF	1	0.92 [0.85–0.99]	0.032	NA
Persistent AF	1	0.28 [0.12–0.66]	0.004	NA
Mixed AF	5	0.91 [0.86–0.97]	0.001	74.30%
Region				
Europe	4	0.89 [0.80–0.99]	0.029	80.50%
North America	1	0.92 [0.85–0.99]	0.032	NA
Asia	2	0.55 [0.17–1.77]	0.317	86.70%
Subgroup	LASr (continuous variable)			
	Study	RR [95% CI]	*P* value	*I* ^2^
**Total**	10	0.93 [0.88–0.99]	0.022	78.6%,
Study design				
Retrospective cohort study	3	1.01 [0.92–1.10]	0.903	87.60%
Prospective cohort study	7	0.89 [0.82–0.96]	0.003	65.10%
Population				
paroxysmal AF	3	0.91 [0.85–0.99]	0.024	25.20%
Persistent AF	1	0.98 [0.91–1.05]	0.508	NA
Mixed AF	6	0.93 [0.85–1.02]	0.102	86.40%
Region				
Europe	3	1.02 [0.93–1.11]	0.653	85.40%
Asia	7	0.88 [0.82–0.95]	0.001	57.60%
Subgroup	PLSct (continuous variable)			
	Study	RR [95% CI]	*P* value	*I* ^2^
**Total**	5	0.97 [0.86–1.09]	0.6	81%
Study design				
Retrospective cohort study	2	0.92 [0.88–0.96]	0.001	0%
Prospective cohort study	3	1.07 [0.74–1.55]	0.719	89.80%
Population				
paroxysmal AF	1	1.22 [0.98–1.52]	0.074	NA
Persistent AF	/	/	/	/
Mixed AF	4	0.92 [0.82–1.04]	0.2	79.60%
Region				
Europe	1	0.94 [0.89–0.98]	0.011	NA
Asia	4	1.01 [0.81–1.24]	0.96	85.60%
Subgroup	LAScd (dichotomous variables)			
	Study	RR [95% CI]	*P* value	*I* ^2^
**Total**	4	0.98 [0.87–1.11]	0.776	94.40%
Study design				
Retrospective cohort study	3	1.03 [0.95–1.12]	0.506	79.40%
Prospective cohort study	1	0.87 [0.83–0.90]	0.001	NA
Region				
Europe	3	1.03 [0.95–1.12]	0.506	79.40%
Asia	1	0.87 [0.83–0.90]	0.001	NA
Subgroup	LAScd (continuous variable)			
	Study	RR [95% CI]	*P* value	*I* ^2^
**Total**	5	0.97 [0.87–1.08]	0.556	79%
Study design				
Retrospective cohort study	1	1.00 [0.96–1.05]	1	NA
Prospective cohort study	4	0.96 [0.81–1.14]	0.656	81.50%
Population				
paroxysmal AF	2	1.10 [0.91–1.32]	0.323	51.60%
Persistent AF	1	0.95 [0.85–1.06]	0.362	NA
Mixed AF	2	0.88 [0.68–1.14]	0.344	92.90%
Region				
Europe	2	0.99 [0.95–1.04]	0.73	0%
Asia	3	0.97 [0.75–1.27]	0.843	87.50%

AF, atrial fibrillation; LASr, reservoir strain; LASct, contraction strain; LAScd, left atrial conduit strain; PALS, peak atrial longitudinal strain; RR, risk ratio; CI, confidence interval.

#### 3.3.2 LASr and AF recurrence

**Figure 4:**
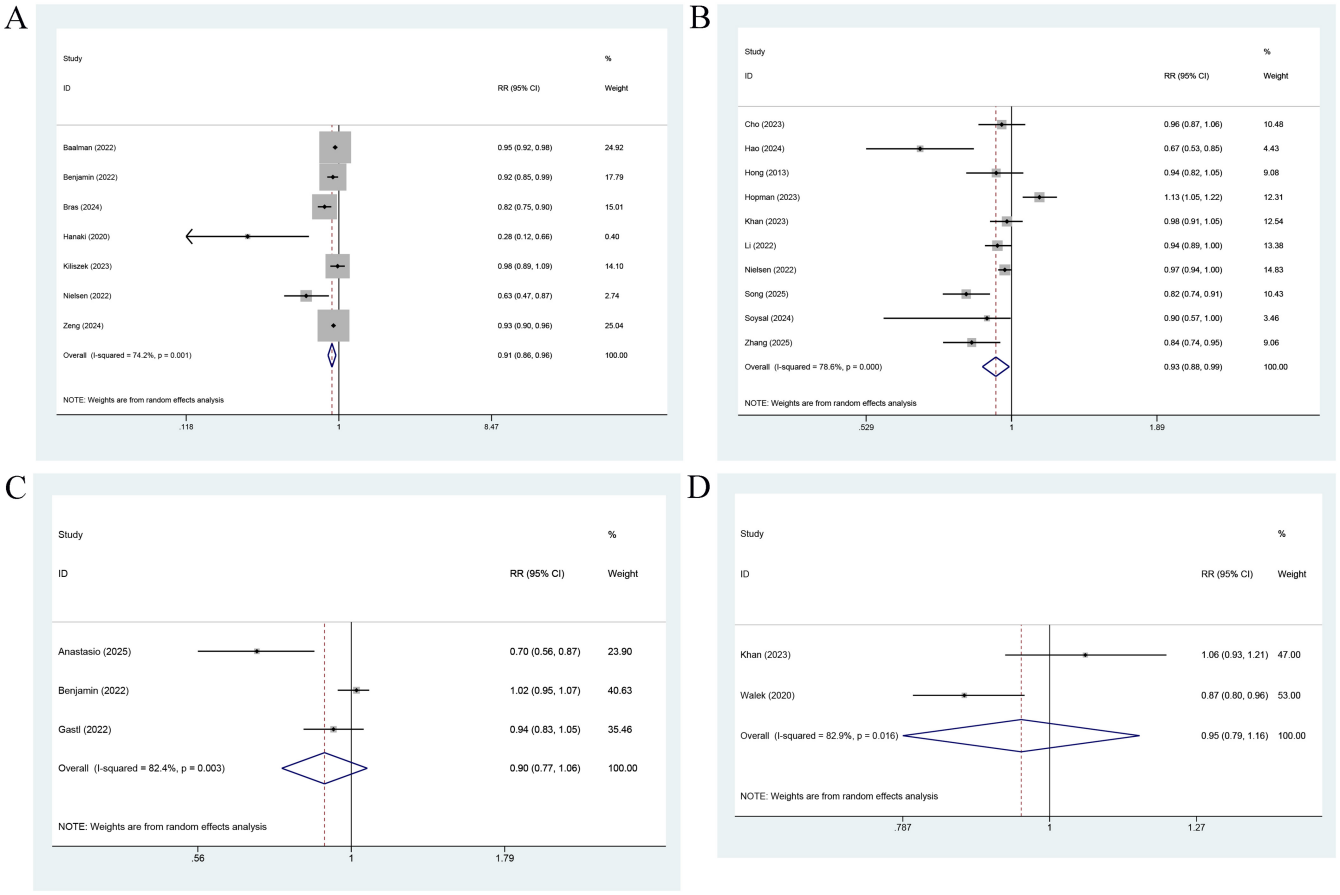
Forest plot for the association between AF recurrence and LASr measured before treatment, analyzed as a categorical variable **(A)** and a continuous variable **(B)**, for the association between AF recurrence and LASr measured after treatment, analyzed as a categorical variable **(C)**, and a continuous variable **(D)**.

LASr measured prior to treatment was pronouncedly linked to AF relapse. Analyzed as a categorical variable [7 studies (15, 16, 21, 25, 31, 35, 39; Figure 4A], a higher LASr was related to a lower risk of AF relapse (RR = 0.91, 95% CI: 0.86–0.96, *P* = 0.001; *I*^2^ = 74.2%). When treated as a continuous variable [10 studies (12, 23, 26–30, 35–37); Figure 4B], a 1-unit elevation in LASr was associated with a roughly 7% lower risk of AF relapse (RR = 0.93, 95% CI: 0.88–0.99, *P* = 0.022; *I*^2^ = 78.6%).

The subgroup analysis demonstrated that the predictive power of LASr measured before treatment was influenced by geographical region, AF type, and study design (Table 2). For LASr as a categorical variable, its predictive performance was significant in North America, Europe, and across all study designs and AF types, but not in Asia. For LASr as a continuous variable, its predictive power was pronounced in Asia, in paroxysmal AF, and in prospective cohort studies, but not in Europe, in persistent or mixed AF, or in retrospective cohort studies.

The meta-regression analysis (Supplementary Table 4) demonstrated that only LVEF was a potential source of heterogeneity in the analysis of LASr as a categorical variable (*P* < 0.05), while demographic and clinical factors were not major sources of heterogeneity in the analysis of LASr as a continuous variable.

By contrast, LASr measured after treatment, whether analyzed as a categorical variable [3 studies (15, 20, 24); Figure 4C] or as a continuous variable [2 studies (29, 38); Figure 4D], showed no significant association with AF relapse (categorical variable: RR = 0.90, 95% CI: 0.77–1.06, *P* = 0.220; continuous variable: RR = 0.95, 95% CI: 0.79–1.16, *P* = 0.638).

#### 3.3.3 LASct and AF recurrence

**Figure 5:**
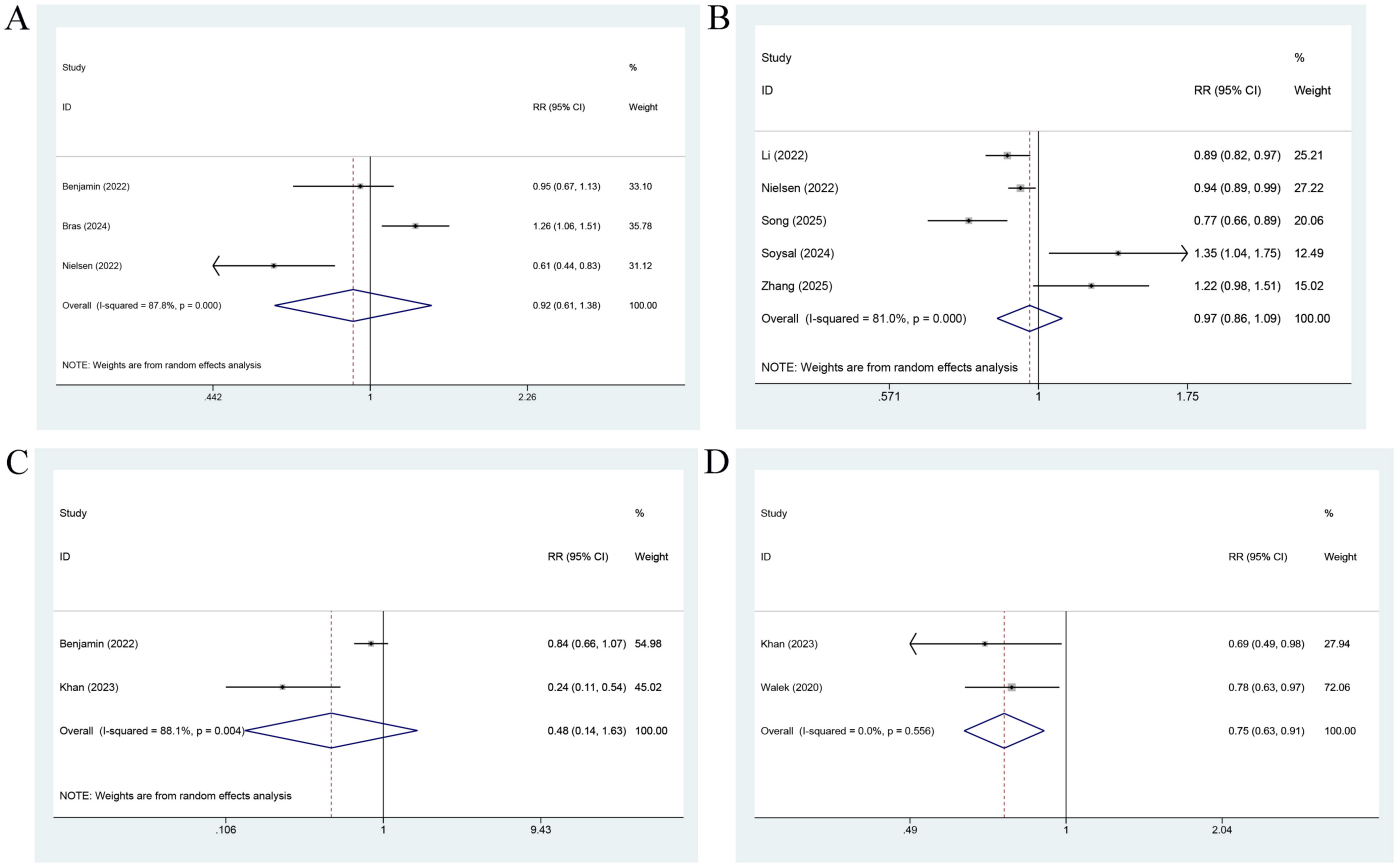
Forest plot for the association between AF recurrence and LASct measured before treatment, analyzed as a categorical variable **(A)**, and a continuous variable **(B)**, for the association between AF recurrence and LASct measured after treatment, analyzed as a categorical variable **(C)**, and a continuous variable **(D)**.

No significant association was observed between LASct measured prior to treatment and AF recurrence, regardless of whether LASct was analyzed as a categorical variable [3 studies (15, 16, 35); Figure 5A] or was treated as a continuous variable [5 studies (12, 30, 35–37); Figure 5B; categorical variable: RR = 0.92, 95% CI: 0.61–1.38, *P* = 0.670; continuous variable: RR = 0.97, 95% CI: 0.86–1.09, *P* = 0.60].

The subgroup analysis indicated that the predictive power of LASct measured before treatment (as a continuous variable) was pronounced in Europe and in retrospective cohort studies (all *P* < 0.05). In contrast, no such predictive power was observed in Asia, across all AF types, or in prospective cohort studies.

When treated as a categorical variable, LASct measured after treatment [2 studies (15, 29); Figure 5C] was not markedly associated with AF relapse (RR = 0.48, 95% CI: 0.14–1.63, *P* = 0.237; *I*^2^ = 88.1%). When analyzed as a continuous variable [2 studies (29, 38); Figure 5D], each unit increase in LASct measured after treatment was associated with an approximately 25% lower risk of AF recurrence (RR = 0.75, 95% CI: 0.63–0.91, *P*=0.002; *I*^2^ = 0%).

#### 3.3.4 LAScd and AF recurrence

**Figure 6:**
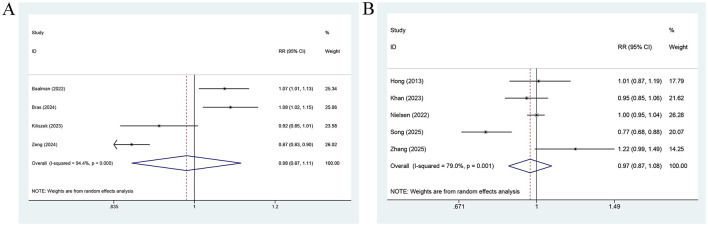
Forest plot for the association between AF recurrence and LAScd measured before treatment, analyzed as a categorical variable **(A)**, and a continuous variable **(B)**.

LAScd measured before treatment was not pronouncedly linked to the risk of AF recurrence. In the 4 studies (16, 21, 31, 39) analyzing LAScd as a categorical variable (Figure 6A), no significant association was observed between a lower LAScd and risk of AF relapse (RR = 0.98, 95% CI: 0.87–1.11, *P* = 0.776). Similarly, in the 5 studies (12, 27, 29, 35, 36) treating LAScd as a continuous variable (Figure 6B), LAScd was not markedly associated with risk of AF recurrence (RR = 0.97, 95% CI: 0.87–1.08, *P* = 0.556). Considerable heterogeneity was present in both analyses (*I*^2^ = 94.4% and 79.0%, respectively). The subgroup analysis suggested that in the analysis of LAScd as a categorical variable, the predictive power of LAScd was pronounced among Asian individuals and in prospective cohorts. However, no such predictive power was observed among European individuals or retrospective cohorts. In contrast, the analysis of LAScd as a continuous variable revealed that the predictive performance of LAScd was not significant for all subgroups (region, AF type, and study design) (Table 2).

### 3.4 Sensitivity analysis

**Figure 7:**
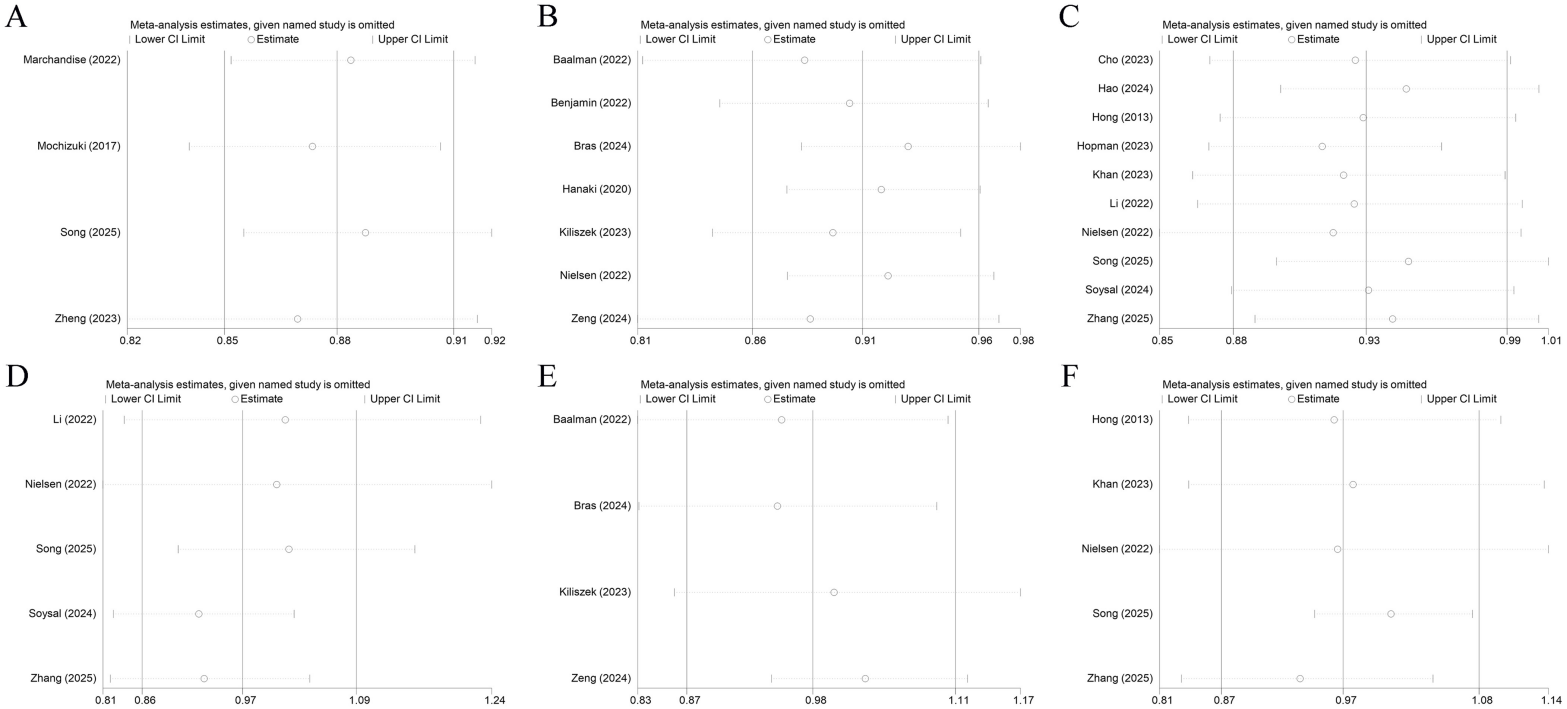
Sensitivity analysis for association between AF recurrence and PALS measured before treatment analyzed as a continuous variable **(A)**, for association between AF recurrence and LASr measured before treatment analyzed as a categorical variable **(B)**, and a continuous variable **(C)**; for association between AF recurrence and LASct measured before treatment analyzed as a continuous variable **(D)**, for association between AF recurrence and LAScd measured before treatment analyzed as a categorical variable **(E)**, and a continuous variable **(F)**.

The sensitivity analysis demonstrated that the results of the meta-analysis were robust (Figures 7A–F). The findings for LASr or LAScd before treatment as a categorical variable and for PALS, LASr, LASct, or LAScd prior to treatment as a continuous variable were not substantially altered by sequentially removing any single study. Sensitivity analysis was not performed for PALS or LASct treated as a categorical variable due to limited data.

### 3.5 Publication bias

Publication bias was evaluated leveraging Egger's test and funnel plots. As shown in Supplementary Figures 1A–F, the funnel plots for PALS, LASr, LASct, and LAScd, all measured before treatment, were generally symmetrical. The results of Egger's test were presented below: PALS measured prior to treatment as a continuous variable (*P* = 0.447); LASr measured before treatment as a categorical variable (*P* = 0.05); LASr measured prior to treatment as continuous variable (*P* = 0.218); LASct measured before treatment as a continuous variable (*P* = 0.505); LAScd measured prior to treatment as a categorical variable (*P* = 0.491); and LAScd measured before treatment as continuous variable (*P* = 0.810).

### 3.6 Meta-analysis of diagnostic test

#### 3.6.1 Threshold effect

The data were imported into Meta DiSc for analysis. Through threshold effect testing for PALS and LASr, we obtained a Spearman's correlation coefficient between the logit of sensitivity and the logit of (1–specificity) of −0.5 (*P* = 0.667) for PALS and that of 0.3 (*P* = 0.624) for LASr, suggesting an absence of a significant threshold effect. Although three studies reported the diagnostic performance of LASct, the pooled analysis revealed a threshold effect (*P* = 0.001). Hence, the pooled specificity and sensitivity for LASct were not calculated.

#### 3.6.2 Pooled effect size of diagnostic test

**Figure 8:**
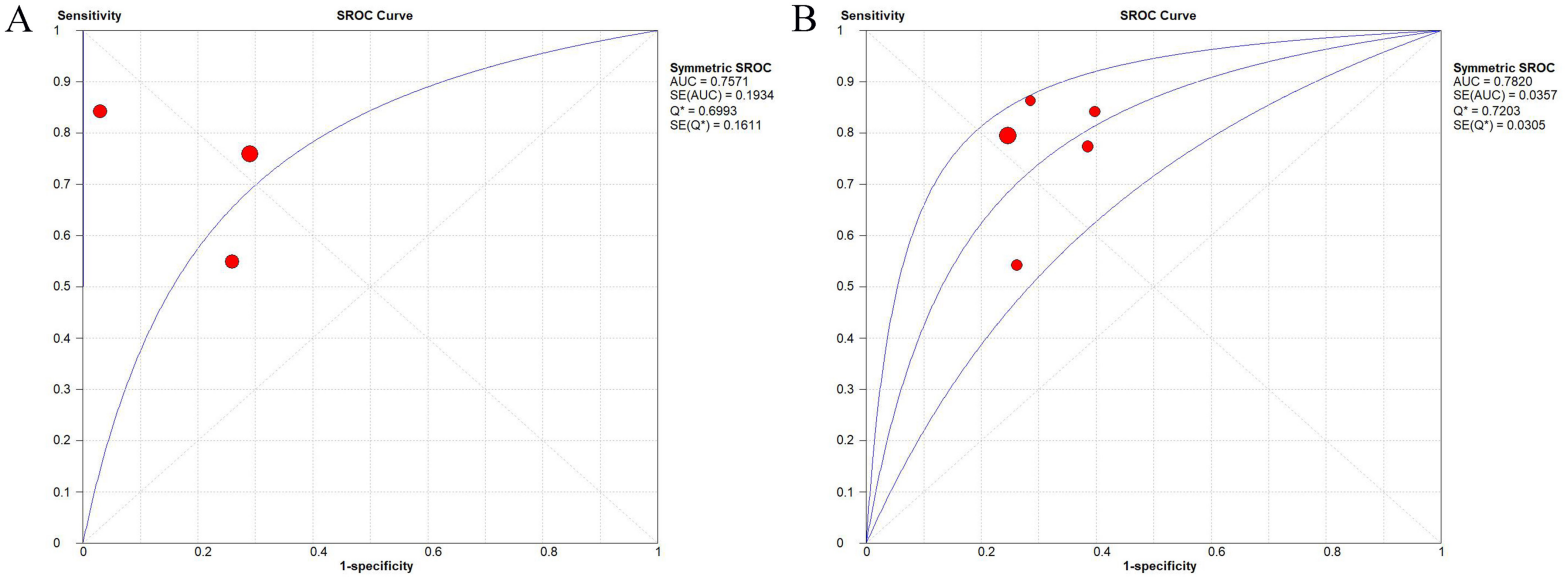
SROC curve for the association between PALS measured before treatment **(A)** or LASr measured before treatment **(B)** and AF recurrence.

Based on the heterogeneity assessment, a bivariate random-effects model was adopted. For PALS measured prior to treatment, the pooled sensitivity, specificity, PLR, NLR, and DOR were 0.75 (95% CI: 0.64–0.84), 0.76 (95% CI: 0.67–0.84), 2.94 (95% CI: 1.25–6.91), 0.33 (95% CI: 0.15–0.72), and 9.99 (95% CI: 2.10–47.63), respectively. For LASr measured before treatment, the pooled sensitivity, specificity, PLR, NLR, and DOR were 0.77 (95% CI: 0.71–0.82), 0.71 (95% CI: 0.66–0.74), 2.49 (95% CI: 1.98–3.14), 0.34 (95% CI: 0.21–0.55), and 7.63 (95% CI: 4.29–13.57), respectively. The details are illustrated in Supplementary Figures 2A–E and Supplementary Figures 3A–E. In the SROC curves, the vertical axis stood for true positive rate, and the horizontal axis represented false positive rate. Figures 8A, B demonstrated that the pooled AUC was 0.75 for PALS and 0.78 for LASr, indicating favorable diagnostic performance of PALS and LASr.

## 4 Discussion

This study comprehensively evaluated the prognostic value of left atrial strain parameters among individuals with AF recurrence. In total, 25 studies involving 3,649 patients were included. This study revealed that both PALS and LASr measured before treatment, whether analyzed as a continuous or categorical variable, were significantly associated with AF recurrence. Although both parameters reflect left atrial reservoir function, they should be interpreted separately due to their distinct origins in ECG triggering: PALS is derived from P-wave triggering, whereas LASr is based on R-wave triggering. Nevertheless, the predictive power of LASct and LAScd was inconsistent. Regarding left atrial strain parameters measured after treatment, most did not reach statistical significance for predicting AF recurrence, except for LASct analyzed as a continuous variable. Furthermore, a diagnostic meta-analysis was performed for the predictive performance of PALS and LASr for risk of AF recurrence. The results revealed pooled AUCs of 0.75 for PALS and 0.78 for LASr, indicating moderate to good discriminatory ability. The findings of this study suggest that both PALS and LASr, as indicators of left atrial reservoir function and compliance, may be applied for risk stratification. Among them, LASr may help identify a subset of patients with potentially high-risk profiles even when traditional structural parameters such as LAVI and LVEF remained relatively preserved. Therefore, PALS and LASr may serve as adjunctive imaging markers for the risk stratification of AF recurrence when interpreted together with conventional clinical and echocardiographic parameters.

From a perspective of physiological mechanism, the results from this study align closely with the distinct roles of the left atrium during different phases of the cardiac cycle. Firstly, both PALS and LASr reflect left atrial reservoir function, defined as the expansion capacity of the left atrium as a receiving chamber during ventricular systole and isovolumic relaxation. However, the two parameters are measured from different ECG reference points: PALS is derived from P-wave triggering, and LASr is measured with R-wave triggering. Consequently, although PALS and LASr describe the same functional phase, they are not fully interchangeable, either in terminology or in the interpretation of absolute values (11, 40). The relevant consensus on atrioventricular deformation imaging in EACVI/ASE and several recent reviews all regard LASr as a core parameter for assessing the function and reconstruction of the left atrium, thereby quantifying the comprehensive damage of each phase of reservoir–conduit–booster (11, 41, 42). Specifically, PALS, using the P-wave as an anchor point, is more sensitive to the expansion of low-voltage regions, signal fractionation, and focal conduction delay. Therefore, PALS identifies potential critical thresholds at the stage when atrial volume has not yet significantly expanded (43). Recent studies also indicate that PALS reflects the compliance and fibrosis of the left atrial myocardium, with lower PALS potentially signifying the subclinical dysfunction of the atrial myocardium. This may be linked to an elevated risk of AF relapse after catheter ablation (14). Moreover, research involving patients undergoing intermediate- to high-risk cardiovascular surgery suggests that a lower LASr is closely correlated with genetically and histologically confirmed atrial fibrotic remodeling. This finding supports the role of LASr as a non-invasive surrogate marker for structural remodeling and interstitial fibrosis (44). Within this physiological framework, long-lasting pressure and volume overload lead to progressive dilation of the left atrium, continuous increase in wall stress, disorientation of atrial muscle fibers, and increased collagen deposition. These alterations result in decreased compliance of the left atrium and impaired reservoir function, manifested as a continuous reduction in the amplitude of reservoir strain at the macroscopic level (45, 46). Reduced LASr is considered a potential marker for the onset, progression, and recurrence of AF (47). Multiple previous cohort studies have consistently shown that patients with reduced PALS or LASr before ablation maintain a significantly heightened risk of AF recurrence after adjusting for other risk factors during the perioperative period. This finding suggests that the impaired function of the left atrial reservoir holds independent prognostic value beyond conventional clinical variables (12, 31, 32). Building upon this evidence, our study further confirmed from a higher evidence-based level that patients with lower PALS and LASr measured before treatment are predisposed to experience arrhythmia recurrence during follow-up, even after undergoing catheter ablation or cardioversion. This finding suggested that the degree of intrinsic structure and electro-mechanical reconstruction, to a considerable extent, determined the long-term effect of strategies for rhythm control.

In contrast, the predictive power of LAScd and LASct was inconsistent in this study, which might be closely related to their physiological meanings and measurement characteristics. LAScd, derived from the negative peak during early diastole in the strain curve, primarily represents the conduit function of the left atrium in early diastole, namely the ability to act as a passive channel between the pulmonary veins and the left ventricle after mitral valve opening. This function is highly dependent on the diastolic function and the instantaneous filling pressure of the left ventricle. Consequently, LAScd is considered a dynamic indicator for left atrium—left ventricle coupling (41, 42). LASct corresponds to the second negative peak in late diastole, reflecting the active ‘booster pump' function of the left atrium under sinus rhythm, the evaluation of which is based on a stable sinus rhythm and intact atrioventricular mechanical coupling (48, 49). Recent reviews and expert consensus have highlighted that conduit and contractile strain components are particularly sensitive to alterations in preload, afterload, heart rate, and diastolic function of the left ventricle, and are more significantly influenced by rhythm status in patients with AF (11, 50, 51). Multiple clinical studies comparing left atrial strain parameters across different rhythm states have shown that LAScd and LASct are significantly lower among individuals with chronic AF compared with those in sinus rhythm. Furthermore, measurements from the same patient exhibit considerable variation between sinus rhythm and AF episodes, which to some extent limits their reproducibility and comparability in real-world settings (52). Additionally, while some follow-up studies before and after catheter ablation reported LASr, LAScd, and LASct, the changes in strain components after catheter ablation were inconsistent, and the relationship of LASr, LAScd, or LASct with AF recurrence was not as stable as that of other electromechanical parameters with AF relapse, suggesting that the signals of catheter phase and contractile response changes in prognosis assessment are easily masked by noise (53). From a technical standpoint, recent guidelines and reviews have repeatedly emphasized that different components of left atrial strain are highly sensitive to image acquisition and analysis methods. Factors such as the selection of ECG trigger (P-wave vs. R-wave), segmentation methods, tracking algorithms, and inconsistencies across different vendor software platforms significantly impact the absolute values and relative changes of LAScd and LASct (41, 54). The superposition of the aforementioned physiological and methodological factors highly possibly dilutes the true association between LAScd/LASct and AF recurrence, thereby resulting in poor effect size and statistical significance in the overall combined analysis of this study.

It is noteworthy that this study found that the predictive value of PALS and LASr measured prior to treatment was the most consistent, whereas the predictive power of LASct and LAScd was less robust overall. Only LASct measured after treatment was significantly associated with AF recurrence. Based on existing research, a plausible explanation is that pre-treatment measurements of both PALS and LASr largely reflect structural or relatively stable remodeling imprints resulting from long-term AF burden, decreased compliance of the left atrium, and interstitial fibrosis (11, 55). Nevertheless, differences in ECG triggering should be accounted for when comparing their absolute values across studies. By contrast, LASct before treatment tends to be affected by factors such as mechanical sluggishness of the atrium and unstable rhythm, contributing to a lower signal-to-noise ratio (56, 57). After treatment, particularly among patients who have successfully restored and maintained sinus rhythm (35), a persistently reduced LASct more directly reflects insufficient contractile reserve of the left atrium and the residual burden of electro-mechanical reconstruction (58). That is, if LASct remains significantly impaired despite temporary rhythm stability, it suggests the left atrial myocardium has difficulty providing an effective ‘booster pump' function, and underlying electrical instability and triggers persist, thereby conferring a higher risk of AF recurrence. This also explains why LASct measured after treatment, which was analyzed as a continuous variable, retained significant predictive capability, while the effects of other parameters were substantially diminished.

In this study, several limitations should be acknowledged. First, all included studies were observational cohort studies, mostly based on single-center data and with a small sample size. Consequently, it was difficult to completely avoid selection bias and residual confounding factors. Therefore, the conclusions primarily indicated association rather than causation. Furthermore, patients in these studies originated from different countries and regions, and the studies were not all conducted across multiple centers, with many based on single-center data. These factors might contribute to unavoidable selection bias and confounding influences. Second, significant heterogeneity was observed in the measurement methods for left atrial strain across different studies, including variations in ultrasound equipment and analysis software, different ECG trigger points (P-wave vs. R-wave), and inconsistent definitions of regions of interest and boundaries. Moreover, a lack of standardization was evident in both the nomenclature and the value assignment for several parameters: PALS and LASr (which both represent reservoir function but are defined using different ECG trigger origins), as well as LAScd and LASct. These differences might affect the accuracy of absolute strain values and effect size estimates. Third, low to moderate degrees of heterogeneity were observed among the included studies. Sample sizes were small, or follow-up durations were inconsistent in certain subgroup analyses, which might have led to the observed heterogeneity and potential publication bias. Although some sources of heterogeneity were identified through meta-regression, the findings in this study remain to be interpreted with caution. Fourth, the limited number of studies for each technical configuration and the diversity of measurement methods posed challenges for performing subgroup analyses, which might also represent a source of the observed heterogeneity. Finally, non-English literature was excluded, with only published English-language studies included, potentially introducing language bias. The inclusion of studies from specific regions or populations might limit the generalizability of the conclusions. In the future, prospective, high-quality, and multicenter clinical trials are warranted to corroborate these findings.

This study also clarified directions for research in the future. First, it is imperative to further standardize the acquisition, nomenclature, and reporting of LAScd and LASct, particularly concerning ECG triggering methods, measurement timepoints, and analysis software. The aim is to clarify their optimal application scenarios in individuals, including but not limited to those with purely paroxysmal AF, patients undergoing first-time ablation, or individuals with multiple comorbid cardiac conditions. Second, it is suggested to explore the integration of multiple left atrial strain parameters with imaging features, biomarkers, and electrophysiological characteristics to construct multimodal prediction models. These efforts hold promise for significantly improving the predictive accuracy for the risk of AF recurrence. In this regard, this study not only corroborated the robust prognostic performance of PALS and LASr as core indicators but also clarified direction and provided theoretical support for subsequent research on optimizing the application of LAScd and LASct and integrating multiple parameters to develop comprehensive prediction tools.

## 5 Conclusion

This study suggests that, among left atrial strain parameters derived from speckle-tracking, both PALS and LASr measured before treatment are associated with a risk of AF recurrence, whereas the overall predictive performance of LAScd and LASct appears limited. PALS and LASr should be regarded as related but distinct reservoir-strain parameters because PALS is based on P-wave triggering and LASr is measured with R-wave triggering. In conclusion, this study suggests that PALS and LASr may be deemed promising imaging biomarkers for the risk stratification of AF recurrence. The combination of either PALS or LASr with conventional clinical and imaging indicators may help optimize an individualized management of patients at risk for AF recurrence.

## Data Availability

The original contributions presented in the study are included in the article/Supplementary material, further inquiries can be directed to the corresponding author.

## References

[1] van Kempen EJ Schellekens MMI Verhoeven JI Ekker MS Verburgt E Immens MHM . Prevalence and factors associated with atrial fibrillation among young patients with ischemic stroke. J Am Heart Assoc. (2025) 14:e043996. doi: 10.1161/JAHA.125.043996 41334730 PMC12826934

[2] Sagris M Vardas EP Theofilis P Antonopoulos AS Oikonomou E Tousoulis D. Atrial fibrillation: pathogenesis, predisposing factors, and genetics. Int J Mol Sci. (2021) 23:6–23. doi: 10.3390/ijms23010006 35008432 PMC8744894

[3] Hindricks G Potpara T Dagres N Arbelo E Bax JJ Blomström-Lundqvist C . Corrigendum to: 2020 ESC guidelines for the diagnosis and management of atrial fibrillation developed in collaboration with the European Association for Cardio-Thoracic Surgery (EACTS): the task force for the diagnosis and management of atrial fibrillation of the European Society of Cardiology (ESC) Developed with the special contribution of the European Heart Rhythm Association (EHRA) of the ESC. Eur Heart J. (2021) 42:4194. doi: 10.1093/eurheartj/ehab648 34520521

[4] Calkins H Hindricks G Cappato R Kim YH Saad EB Aguinaga L . 2017 HRS/EHRA/ECAS/APHRS/SOLAECE expert consensus statement on catheter and surgical ablation of atrial fibrillation. Heart Rhythm. (2017) 14:e275–444. doi: 10.1016/j.hrthm.2017.05.012 28506916 PMC6019327

[5] Nie L Zhang T Wang W Han X Liu M Zhang S . Machine learning-based prediction model for recurrence after radiofrequency catheter ablation in patients with atrial fibrillation. Front Cardiovasc Med. (2025) 12:1642409. doi: 10.3389/fcvm.2025.1642409 40860368 PMC12370652

[6] Dretzke J Chuchu N Agarwal R Herd C Chua W Fabritz L . Predicting recurrent atrial fibrillation after catheter ablation: a systematic review of prognostic models. Europace. (2020) 22:748–60. doi: 10.1093/europace/euaa041 32227238 PMC7203634

[7] Fabritz L Hatem SN Sossalla S. Arrhythmia-induced cardiomyopathy: focus on atrial fibrillation. Nat Rev Cardiol. (2025) 23:197–207. doi: 10.1038/s41569-025-01195-2 40954334

[8] Dzeshka MS Lip GY Snezhitskiy V Shantsila E. Cardiac fibrosis in patients with atrial fibrillation: mechanisms and clinical implications. J Am Coll Cardiol. (2015) 66:943–59. doi: 10.1016/j.jacc.2015.06.1313 26293766

[9] Daise MA Maule G Ismail M Alqudah Q Mojaddedi S Obeidat O . Atrial cardiomyopathy: current clinical perspectives and future insights. Future Cardiol. (2025) 21:1097–105. doi: 10.1080/14796678.2025.2548159 40811051 PMC12854605

[10] Moreno-Ruiz LA Madrid-Miller A Martínez-Flores JE González-Hermosillo JA Arenas-Fonseca J Zamorano-Velázquez N . Left atrial longitudinal strain by speckle tracking as independent predictor of recurrence after electrical cardioversion in persistent and long standing persistent non-valvular atrial fibrillation. Int J Cardiovasc Imaging. (2019) 35:1587–96. doi: 10.1007/s10554-019-01597-7 30993507 PMC6700045

[11] Rusali CA Lupu IC Rusali LM Cojocaru L. Left atrial strain-current review of clinical applications. Diagnostics. (2025) 15:1347–4370. doi: 10.3390/diagnostics15111347 40506919 PMC12154511

[12] Zhang R Li H Wang Y Yu T Li J Wu Y . Left atrial strain predicts paroxysmal atrial fibrillation recurrence after catheter ablation: a 1-year study using three-dimensional speckle-tracking echocardiography. BMC Cardiovasc Disord. (2025) 25:78. doi: 10.1186/s12872-024-04447-0 39905319 PMC11792397

[13] Knappe D Vogler J Weimann J Banas V Obergassel J Yildirim S . Left atrial reservoir strain and recurrence of atrial fibrillation following *de-novo* pulmonary vein isolation - results of the ASTRA-AF pilot study. Circ J. (2025) 89:153–61. doi: 10.1253/circj.CJ-24-0209 38839350

[14] Zheng D Zhang Y Huang D Wang M Guo N Zhu S . Incremental predictive utility of a radiomics signature in a nomogram for the recurrence of atrial fibrillation. Front Cardiovasc Med. (2023) 10:1203009. doi: 10.3389/fcvm.2023.1203009 37636308 PMC10451088

[15] Benjamin MM Moulki N Waqar A Ravipati H Schoenecker N Wilber D . Association of left atrial strain by cardiovascular magnetic resonance with recurrence of atrial fibrillation following catheter ablation. J Cardiovasc Magn Reson. (2022) 24:3. doi: 10.1186/s12968-021-00831-3 34980165 PMC8722067

[16] Brás PG Cunha PS Timóteo AT Portugal G Galrinho A Laranjo S . Evaluation of left atrial strain imaging and integrated backscatter as predictors of recurrence in patients with paroxysmal, persistent, and long-standing persistent atrial fibrillation undergoing catheter ablation. J Interv Card Electrophysiol. (2024) 67:479–92. doi: 10.1007/s10840-023-01602-z 37414922

[17] Page MJ McKenzie JE Bossuyt PM Boutron I Hoffmann TC Mulrow CD . The PRISMA 2020 statement: an updated guideline for reporting systematic reviews. Bmj. (2021) 372:n71. doi: 10.1136/bmj.n71 33782057 PMC8005924

[18] Wade R Corbett M Eastwood A. Quality assessment of comparative diagnostic accuracy studies: our experience using a modified version of the QUADAS-2 tool. Res Synth Methods. (2013) 4:280–6. doi: 10.1002/jrsm.1080 26053845

[19] Stang A. Critical evaluation of the Newcastle-Ottawa scale for the assessment of the quality of nonrandomized studies in meta-analyses. Eur J Epidemiol. (2010) 25:603–5. doi: 10.1007/s10654-010-9491-z 20652370

[20] Anastasio F Pastorini G Pucci G Gonella A Tardivo V Feola M. Predicting early atrial fibrillation recurrence post-electrical cardioversion: a critical look at bilateral atrial function. J Clin Med. (2025) 14:749–60. doi: 10.3390/jcm14030749 39941419 PMC11818095

[21] Baalman SWE van den Berg NWE Neefs J Berger WR Meulendijks ER de Bruin-Bon R . Left atrial strain and recurrence of atrial fibrillation after thoracoscopic surgical ablation: a subanalysis of the AFACT study. Int J Cardiovasc Imaging. (2022) 38:2615–24. doi: 10.1007/s10554-022-02645-5 36445663 PMC9708776

[22] Bai Y Zhao Y Li J Zhang Y Bai R Du X . Association of peak atrial longitudinal strain with atrial fibrillation recurrence in patients with chronic lung diseases following radiofrequency ablation. Intern Med J. (2018) 48:851–9. doi: 10.1111/imj.13768 29460463

[23] Cho DH Kim YG Choi J Kim HD Kim MN Shim J . Atrial cardiomyopathy with impaired functional reserve in patients with paroxysmal atrial fibrillation. J Am Soc Echocardiogr. (2023) 36:180–8. doi: 10.1016/j.echo.2022.09.012 36162771

[24] Gastl M Bejinariu A Behm P Lindert A Kelm M Makimoto H . Role of CMR-derived atrial deformation analysis in the prediction of atrial fibrillation recurrence rate after pulmonary vein isolation. Eur J Radiol. (2022) 155:110452. doi: 10.1016/j.ejrad.2022.110452 35952478

[25] Hanaki Y Machino-Ohtsuka T Aonuma K Komatsu Y Machino T Yamasaki H . Preprocedural restoration of sinus rhythm and left atrial strain predict outcomes of catheter ablation for long-standing persistent atrial fibrillation. J Cardiovasc Electrophysiol. (2020) 31:1709–18. doi: 10.1111/jce.14540 32391641

[26] Hao X Li W Zhang Q Cao L Wang J Guo L . Assessing left atrial appendage functions by transesophageal echocardiography and speckle tracking imaging to predict recurring atrial fibrillation post-radiofrequency catheter ablation. Echocardiography. (2024) 41:e15958. doi: 10.1111/echo.15958 39403006

[27] Hong J Gu X An P Luo T Lv Q Kang J . Left atrial functional remodeling in lone atrial fibrillation: a two-dimensional speckle tracking echocardiographic study. Echocardiography. (2013) 30:1051–60. doi: 10.1111/echo.12200 23557171

[28] Hopman L Mulder MJ van der Laan AM Bhagirath P Demirkiran A von Bartheld MB . Left atrial strain is associated with arrhythmia recurrence after atrial fibrillation ablation: cardiac magnetic resonance rapid strain vs. feature tracking strain. Int J Cardiol. (2023) 378:23–31. doi: 10.1016/j.ijcard.2023.02.019 36804765

[29] Khan HR Yakupoglu HY Kralj-Hans I Haldar S Bahrami T Clague J . Left atrial function predicts atrial arrhythmia recurrence following ablation of long-standing persistent atrial fibrillation. Circ Cardiovasc Imaging. (2023) 16:e015352. doi: 10.1161/CIRCIMAGING.123.015352 37288553 PMC10281195

[30] Li Y Li Y Sun L Ye X Cai Q Zhu W . Left atrial strain for predicting recurrence in patients with non-valvular atrial fibrillation after catheter ablation: a single-center two-dimensional speckle tracking retrospective study. BMC Cardiovasc Disord. (2022) 22:468. doi: 10.1186/s12872-022-02916-y 36335294 PMC9637312

[31] Kiliszek M Uziebło-Życzkowska B Krzyżanowski K Jurek A Wierzbowski R Smalc-Stasiak M . Value of left atrial strain in predicting recurrence after atrial fibrillation ablation. J Clin Med. (2023) 12:4034–44. doi: 10.3390/jcm12124034 37373726 PMC10299493

[32] Marchandise S Garnir Q Scavée C Varnavas V le Polain de Waroux JB Wauters A . Prediction of left atrial fibrosis and success of catheter ablation by speckle tracking echocardiography in patients imaged in persistent atrial fibrillation. Front Cardiovasc Med. (2022) 9:856796. doi: 10.3389/fcvm.2022.856796 35694674 PMC9176405

[33] Mochizuki A Yuda S Fujito T Kawamukai M Muranaka A Nagahara D . Left atrial strain assessed by three-dimensional speckle tracking echocardiography predicts atrial fibrillation recurrence after catheter ablation in patients with paroxysmal atrial fibrillation. J Echocardiogr. (2017) 15:79–87. doi: 10.1007/s12574-017-0329-5 28155065

[34] Motoc A Luchian ML Scheirlynck E Roosens B Chameleva H Gevers M . Incremental value of left atrial strain to predict atrial fibrillation recurrence after cryoballoon ablation. PLoS ONE. (2021) 16:e0259999. doi: 10.1371/journal.pone.0259999 34797844 PMC8604362

[35] Nielsen AB Skaarup KG Djernæs K Hauser R San José Estépar R Sørensen SK . Left atrial contractile strain predicts recurrence of atrial tachyarrhythmia after catheter ablation. Int J Cardiol. (2022) 358:51–7. doi: 10.1016/j.ijcard.2022.04.056 35469934

[36] Song Y Huang L Jiang C Du F Zhang J Chang P. Usefulness of two-dimensional speckle tracking echocardiography in assessment of left atrial fibrosis degree and its application in atrial fibrillation. Int J Cardiovasc Imaging. (2025) 41:695–708. doi: 10.1007/s10554-025-03345-6 40063159 PMC11982115

[37] Soysal AU Gulfidan A Raimoglou D Atici A Yalman H Kucur M . Comprehensive analysis of recurrence factors in cryoballoon AF ablation: integrating clinical, biomarkers, and echocardiographic parameters. Int J Cardiovasc Imaging. (2024) 40:2271–81. doi: 10.1007/s10554-024-03218-4 39147919

[38] Wałek P Ciesla E Gorczyca I Wożakowska-Kapłon Wożakowska-Kapłon B. Left atrial wall dyskinesia assessed during contractile phase as a predictor of atrial fibrillation recurrence after electrical cardioversion performed due to persistent atrial fibrillation. Medicine. (2020) 99:e23333. doi: 10.1097/MD.0000000000023333 33285712 PMC7717756

[39] Zeng D Li L Chang S Zhang X Zhong Y Cai Y . The utility of speckle tracking echocardiographic parameters in predicting atrial fibrillation recurrence after catheter ablation in patients with non-valvular atrial fibrillation. Ther Clin Risk Manag. (2024) 20:719–29. doi: 10.2147/TCRM.S486056 39386226 PMC11463183

[40] Goyal A Abbasi HQ Yakkali S Khan AM Tariq MD Sohail AH . Left atrial strain as a predictor of early anthracycline-induced chemotherapy-related cardiac dysfunction: a pilot systematic review and meta-analysis. J Clin Med. (2024) 13:3904–15. doi: 10.3390/jcm13133904 38999470 PMC11242155

[41] Badano LP Kolias TJ Muraru D Abraham TP Aurigemma G Edvardsen T . Standardization of left atrial, right ventricular, and right atrial deformation imaging using two-dimensional speckle tracking echocardiography: a consensus document of the EACVI/ASE/Industry Task Force to standardize deformation imaging. Eur Heart J Cardiovasc Imaging. (2018) 19:591–600. doi: 10.1093/ehjci/jey042 29596561

[42] Silva MR Sampaio F Braga J Ribeiro J Fontes-Carvalho R. Left atrial strain evaluation to assess left ventricle diastolic dysfunction and heart failure with preserved ejection fraction: a guide to clinical practice: left atrial strain and diastolic function. Int J Cardiovasc Imaging. (2023) 39:1083–96. doi: 10.1007/s10554-023-02816-y 36826616

[43] Seewoester T Nedios S Dagres N Marinov K Hindricks G Bollmann A . The novel P-Wave Area index predicts low voltage areas in patients undergoing pulmonary vein isolation for treatment of atrial fibrillation. Eur Heart J. (2023) 44:ehad655.567. doi: 10.1093/eurheartj/ehad655.567

[44] Nakajima T Haruyama A Fukuda T Minami K Hirose S Yazawa H . Left atrial reservoir strain is a marker of atrial fibrotic remodeling in patients undergoing cardiovascular surgery: analysis of gene expression. PLoS ONE. (2024) 19:e0306323. doi: 10.1371/journal.pone.0306323 38976680 PMC11230549

[45] Henein MY Holmgren A Lindqvist P. Left atrial function in volume versus pressure overloaded left atrium. Int J Cardiovasc Imaging. (2015) 31:959–65. doi: 10.1007/s10554-015-0638-6 25759088

[46] Hunter RJ Liu Y Lu Y Wang W Schilling RJ. Left atrial wall stress distribution and its relationship to electrophysiologic remodeling in persistent atrial fibrillation. Circ Arrhythm Electrophysiol. (2012) 5:351–60. doi: 10.1161/CIRCEP.111.965541 22294615

[47] Serenelli M Cantone A Dal Passo B Di Ienno L Fiorio A Pavasini R . Atrial longitudinal strain predicts new-onset atrial fibrillation: a systematic review and meta-analysis. JACC Cardiovasc Imaging. (2023) 16:392–5. doi: 10.1016/j.jcmg.2022.11.004 36648050

[48] Gan GCH Ferkh A Boyd A Thomas L. Left atrial function: evaluation by strain analysis. Cardiovasc Diagn Ther. (2018) 8:29–46. doi: 10.21037/cdt.2017.06.08 29541609 PMC5835645

[49] White WL. Erratum to: why i hate the index finger. Hand. (2011) 6:233. doi: 10.1007/s11552-011-9321-0 21776199 PMC3092884

[50] Nagueh SF Khan SU. Left atrial strain for assessment of left ventricular diastolic function: focus on populations with normal LVEF. JACC Cardiovasc Imaging. (2023) 16:691–707. doi: 10.1016/j.jcmg.2022.10.011 36752445

[51] Brian DH. Evaluation of left atrial function: current status. Structural Heart. (2017) 1:109–20. doi: 10.1080/24748706.2017.1353718

[52] Hwozdyk L Cudd TH Anatoliotakis N Junejo RT. Using left atrial strain values and correlating changes in left atrium area and volume size as an early predictor of atrial fibrillation: a prospective, single-centre case-control study. Eur Heart J Cardiovasc Imaging. (2025) 26:jeae333.286. doi: 10.1093/ehjci/jeae333.286

[53] Angelini E Sieweke JT Berliner D Biber S Hohmann S Oldhafer M . Echocardiographic parameters indicating left atrial reverse remodeling after catheter ablation for atrial fibrillation. Front Cardiovasc Med. (2023) 10:1270422. doi: 10.3389/fcvm.2023.1270422 38164465 PMC10757954

[54] Javadi N Bismee NN Abbas MT Scalia IG Pereyra M Baba Ali N . Left atrial strain: state of the art and clinical implications. J Pers Med. (2024) 14:1093–110. doi: 10.3390/jpm14111093 39590585 PMC11595645

[55] Inoue K Smiseth OA. Left atrium as key player and essential biomarker in heart failure. J Cardiol. (2025) 85:8–16. doi: 10.1016/j.jjcc.2024.07.006 39084316

[56] O'Neill T Kang P Hagendorff A Tayal B. The clinical applications of left atrial strain: a comprehensive review. Medicina. (2024) 60:693–713. doi: 10.3390/medicina60050693 38792875 PMC11123486

[57] Inoue K Obokata M. Clinical utility of the left atrial strain analysis. J Echocardiogr. (2025) 23:145–55. doi: 10.1007/s12574-025-00695-x 40608278 PMC12378494

[58] Tantawi S Issa E Matli K Farah R Costanian C Miner S . Diagnostic and prognostic potential of left atrial strain in cardiovascular disease: a narrative review. J Echocardiogr. (2025) 23:69–85. doi: 10.1007/s12574-024-00677-5 39731693

