# Supplementary material for: Predictive accuracy of left atrial strain parameters for risk of atrial fibrillation recurrence: a systematic review and meta-analysis
Source: Front Med (Lausanne). 2026 Apr 20;13:1798092. doi: 10.3389/fmed.2026.1798092 (PMC13136001; doi:10.3389/fmed.2026.1798092)
Supplement: Supplementary file 1 [file Data_Sheet_1.docx]

**Supplementary Materials**

**Supplementary Table 1.** Search strategy.

| PUBMED |
| --- |
| ((Atrial Fibrillation[MeSH Terms]) OR (Atrial Fibrillation[Title/Abstract] OR Atrial Fibrillations[Title/Abstract] OR Fibrillation, Atrial[Title/Abstract] OR Fibrillations, Atrial[Title/Abstract] OR Auricular Fibrillation[Title/Abstract] OR Auricular Fibrillations[Title/Abstract] OR Fibrillation, Auricular[Title/Abstract] OR Fibrillations, Auricular[Title/Abstract] OR Persistent Atrial Fibrillation[Title/Abstract] OR Atrial Fibrillation, Persistent[Title/Abstract] OR Atrial Fibrillations, Persistent[Title/Abstract] OR Fibrillation, Persistent Atrial[Title/Abstract] OR Fibrillations, Persistent Atrial[Title/Abstract] OR Persistent Atrial Fibrillations[Title/Abstract] OR Familial Atrial Fibrillation[Title/Abstract] OR Atrial Fibrillation, Familial[Title/Abstract] OR Atrial Fibrillations, Familial[Title/Abstract] OR Familial Atrial Fibrillations[Title/Abstract] OR Fibrillation, Familial Atrial[Title/Abstract] OR Fibrillations, Familial Atrial[Title/Abstract] OR Paroxysmal Atrial Fibrillation[Title/Abstract] OR Atrial Fibrillation, Paroxysmal[Title/Abstract] OR Atrial Fibrillations, Paroxysmal[Title/Abstract] OR atrium fibrillation[Title/Abstract] OR auricular fibrilation[Title/Abstract] OR cardiac atrial fibrillation[Title/Abstract] OR cardiac atrium fibrillation[Title/Abstract] OR fibrillation, heart atrium[Title/Abstract] OR Fibrillation, Paroxysmal Atrial[Title/Abstract] OR Fibrillations, Paroxysmal Atrial[Title/Abstract] OR heart atrial fibrillation[Title/Abstract] OR heart atrium fibrillation[Title/Abstract] OR heart fibrillation atrium[Title/Abstract] OR nonvalvular atrial fibrillation[Title/Abstract] OR non-valvular atrial fibrillation[Title/Abstract] OR Paroxysmal Atrial Fibrillations[Title/Abstract])) AND (left atrial strain[Title/Abstract] OR LA strain[Title/Abstract] OR atrial deformation[Title/Abstract] OR atrial mechanics[Title/Abstract] OR atrial stiffness[Title/Abstract] OR atrial reservoir strain[Title/Abstract] OR atrial contractile strain[Title/Abstract] OR atrial conduit strain[Title/Abstract] OR left atrial function[Title/Abstract] OR atrial longitudinal strain[Title/Abstract] OR Peak Atrial Longitudinal Strain[Title/Abstract] OR Left Atrial Reservoir Strain[Title/Abstract] OR Left Atrial Contraction Strain[Title/Abstract]) |
| Embase |
| ('passive filling phase strain left atrial strain':ab,ti OR 'la strain':ab,ti OR 'left atrium strain':ab,ti OR 'atrial deformation':ab,ti OR 'la reservoir strain':ab,ti OR 'la global longitudinal strain':ab,ti OR 'atrial longitudinal strain':ab,ti OR 'left atrial longitudinal strain':ab,ti OR 'peak reservoir strain':ab,ti OR 'la total strain':ab,ti OR 'la storage strain':ab,ti OR 'atrial contractile strain':ab,ti OR 'passive filling phase strain':ab,ti OR 'la conduit strain':ab,ti OR 'passive emptying strain':ab,ti OR 'atrial conduit strain':ab,ti OR 'early diastolic la strain':ab,ti OR 'la passive strain':ab,ti OR 'conduit function strain':ab,ti OR 'la contractile strain':ab,ti OR 'la booster strain':ab,ti OR 'active emptying strain':ab,ti OR 'la pump strain':ab,ti OR 'atrial systole strain':ab,ti OR 'booster pump function strain':ab,ti) AND ('atrial fibrillation':ab,ti OR 'atrial fibrillations':ab,ti OR 'fibrillation, atrial':ab,ti OR 'fibrillations, atrial':ab,ti OR 'auricular fibrillation':ab,ti OR 'auricular fibrillations':ab,ti OR 'fibrillation, auricular':ab,ti OR 'fibrillations, auricular':ab,ti OR 'persistent atrial fibrillation':ab,ti OR 'atrial fibrillation, persistent':ab,ti OR 'atrial fibrillations, persistent':ab,ti OR 'fibrillation, persistent atrial':ab,ti OR 'fibrillations, persistent atrial':ab,ti OR 'persistent atrial fibrillations':ab,ti OR 'familial atrial fibrillation':ab,ti OR 'atrial fibrillation, familial':ab,ti OR 'atrial fibrillations, familial':ab,ti OR 'familial atrial fibrillations':ab,ti OR 'fibrillation, familial atrial':ab,ti OR 'fibrillations, familial atrial':ab,ti OR 'paroxysmal atrial fibrillation':ab,ti OR 'atrial fibrillation, paroxysmal':ab,ti OR 'atrial fibrillations, paroxysmal':ab,ti OR 'atrium fibrillation':ab,ti OR 'auricular fibrilation':ab,ti OR 'cardiac atrial fibrillation':ab,ti OR 'cardiac atrium fibrillation':ab,ti OR 'fibrillation, heart atrium':ab,ti OR 'fibrillation, paroxysmal atrial':ab,ti OR 'fibrillations, paroxysmal atrial':ab,ti OR 'heart atrial fibrillation':ab,ti OR 'heart atrium fibrillation':ab,ti OR 'heart fibrillation atrium':ab,ti OR 'nonvalvular atrial fibrillation':ab,ti OR 'non-valvular atrial fibrillation':ab,ti OR 'paroxysmal atrial fibrillations':ab,ti OR 'atrial fibrillation'/exp) |
| Cochrane |
| (((Atrial Fibrillation OR Atrial Fibrillations OR Fibrillation, Atrial OR Fibrillations, Atrial OR Auricular Fibrillation OR Auricular Fibrillations OR Fibrillation, Auricular OR Fibrillations, Auricular OR Persistent Atrial Fibrillation OR Atrial Fibrillation, Persistent OR Atrial Fibrillations, Persistent OR Fibrillation, Persistent Atrial OR Fibrillations, Persistent Atrial OR Persistent Atrial Fibrillations OR Familial Atrial Fibrillation OR Atrial Fibrillation, Familial OR Atrial Fibrillations, Familial OR Familial Atrial Fibrillations OR Fibrillation, Familial Atrial OR Fibrillations, Familial Atrial OR Paroxysmal Atrial Fibrillation OR Atrial Fibrillation, Paroxysmal OR Atrial Fibrillations, Paroxysmal OR atrium fibrillation OR auricular fibrilation OR cardiac atrial fibrillation OR cardiac atrium fibrillation OR fibrillation, heart atrium OR Fibrillation, Paroxysmal Atrial OR Fibrillations, Paroxysmal Atrial OR heart atrial fibrillation OR heart atrium fibrillation OR heart fibrillation atrium OR nonvalvular atrial fibrillation OR non-valvular atrial fibrillation OR Paroxysmal Atrial Fibrillations):ti,ab,kw) OR MeSH descriptor: [Atrial Fibrillation] explode all trees) AND ((LA reservoir strain OR LA global longitudinal strain OR left atrial longitudinal strain OR peak reservoir strain OR LA total strain OR LA storage strain OR passive filling phase strain left atrial strain OR LA strain OR left atrium strain OR atrial deformation OR LA reservoir strain OR LA global longitudinal strain OR atrial longitudinal strain OR left atrial longitudinal strain OR peak reservoir strain OR LA total strain OR LA storage strain OR atrial contractile strain OR passive filling phase strain OR LA conduit strain OR passive emptying strain OR atrial conduit strain OR early diastolic LA strain OR LA passive strain OR conduit function strain OR LA contractile strain OR LA booster strain OR active emptying strain OR LA pump strain OR atrial systole strain OR booster pump function strain):ti,ab,kw) |
| Web of science |
| "Atrial Fibrillation" OR "Atrial Fibrillations" OR "Fibrillation, Atrial" OR "Fibrillations, Atrial" OR "Auricular Fibrillation" OR "Auricular Fibrillations" OR "Fibrillation, Auricular" OR "Fibrillations, Auricular" OR "Persistent Atrial Fibrillation" OR "Atrial Fibrillation, Persistent" OR "Atrial Fibrillations, Persistent" OR "Fibrillation, Persistent Atrial" OR "Fibrillations, Persistent Atrial" OR "Persistent Atrial Fibrillations" OR "Familial Atrial Fibrillation" OR "Atrial Fibrillation, Familial" OR "Atrial Fibrillations, Familial" OR "Familial Atrial Fibrillations" OR "Fibrillation, Familial Atrial" OR "Fibrillations, Familial Atrial" OR "Paroxysmal Atrial Fibrillation" OR "Atrial Fibrillation, Paroxysmal" OR "Atrial Fibrillations, Paroxysmal" OR "atrium fibrillation" OR "auricular fibrilation" OR "cardiac atrial fibrillation" OR "cardiac atrium fibrillation" OR "fibrillation, heart atrium" OR "Fibrillation, Paroxysmal Atrial" OR "Fibrillations, Paroxysmal Atrial" OR "heart atrial fibrillation" OR "heart atrium fibrillation" OR "heart fibrillation atrium" OR "nonvalvular atrial fibrillation" OR "non-valvular atrial fibrillation" OR "Paroxysmal Atrial Fibrillations" (Topic) and "LA reservoir strain" OR "LA global longitudinal strain" OR "left atrial longitudinal strain" OR "peak reservoir strain" OR "LA total strain" OR "LA storage strain" OR "passive filling phase strain left atrial strain" OR "LA strain" OR "left atrium strain" OR "atrial deformation" OR "LA reservoir strain" OR "LA global longitudinal strain" OR "atrial longitudinal strain" OR "left atrial longitudinal strain" OR "peak reservoir strain" OR "LA total strain" OR "LA storage strain" OR "atrial contractile strain" OR "passive filling phase strain" OR "LA conduit strain" OR "passive emptying strain" OR "atrial conduit strain" OR "early diastolic LA strain" OR "LA passive strain" OR "conduit function strain" OR "LA contractile strain" OR "LA booster strain" OR "active emptying strain" OR "LA pump strain" OR "atrial systole strain" OR "booster pump function strain" (Topic) and Preprint Citation Index (Exclude – Database) |

**Supplementary Table 2.** The mean plus standard deviation of left atrial strain parameters before and after treatment for AF recurrence

| **Pre-treatment LASr** | **Author** | **Year** | **Recurrence** | **No recurrence** |
| --- | --- | --- | --- | --- |
|  | Baalman | 2022 | 13±10.2 | 16.6±13.6 |
|  | Bras | 2024 | 9.8±5.8 | 23.2±9.8 |
|  | Cho | 2023 | NR | NR |
|  | Hanaki | 2020 | 8±2.4 | 8.9±2.8 |
|  | Hao | 2024 | 13.53±1.51 | 18.26±4.46 |
|  | Hong | 2013 | NR | NR |
|  | Hopman | 2023 | -14.67±4.49 | -16.97±3.47 |
|  | Khan | 2023 | 10.4±4.1 | 11.1±3.9 |
|  | Li | 2022 | 16.6±7.3 | 20.4±8.4 |
|  | Kiliszek | 2023 | NR | NR |
|  | Nielsen | 2022 | 22.92±7.97 | 25.14±8.41 |
|  | Song | 2025 | 6.54±4 | 15.52±9.32 |
|  | Soysal | 2024 | 22.3±13.3 | 30.5±12.4 |
|  | Zeng | 2024 | 17.02±5.89 | 24.63±7.28 |
|  | Zhang | 2025 | 14.2±4.2 | 17.07±1.45 |
| **Post-treatment LASr** | **Author** | **Year** | **Recurrence** | **No recurrence** |
|  | Anastasio | 2025 | 9±4.2 | 15.8±7.7 |
|  | Benjamin | 2022 | 20.8±8.9 | 25.2±10.7 |
|  | Benjamin | 2022 | 21±9.1 | 21.4±7.8 |
|  | Gastl | 2022 | 10.18±4.88 | 12.63±6.72 |
|  | Khan | 2023 | 16.8±5.2 | 20.8±6.1 |
|  | Soysal | 2024 | 24.2±13.4 | 37.3±12 |
|  | Walek | 2020 | 11.22±3.93 | 14.68±5.33 |
|  | Zhang | 2025 | 14±3.8 | 20.93±1.45 |
| **Pre-treatment LAScd** | **Author** | **Year** | **Recurrence** | **No recurrence** |
|  | Baalman | 2022 | -9±5.3 | -11.8±7.5 |
|  | Bras | 2024 | -6.7±4.1 | -12.2±6.7 |
|  | Hong | 2013 | NR | NR |
|  | Khan | 2023 | 5.2±2.1 | 5.9±2.7 |
|  | Kiliszek | 2023 | NR | NR |
|  | Nielsen | 2022 | 12.49±5.44 | 13.26±5.13 |
|  | Song | 2025 | 4.29±2.97 | 9.06±5.22 |
|  | Zeng | 2024 | 6.96±4.41 | 13.29±6.97 |
|  | Zhang | 2025 | -8.6±2.6 | -9.02±0.89 |
| **Post-treatment LAScd** | **Author** | **Year** | **Recurrence** | **No recurrence** |
|  | Gastl | 2022 | 7.72±4.22 | 7.66±5.52 |
|  | Khan | 2023 | 10.2±4.1 | 12.3±4.6 |
|  | Zhang | 2025 | -8.9±3.1 | -11.07±1.04 |
| **Pre-treatment LASct** | **Author** | **Year** | **Recurrence** | **No recurrence** |
|  | Benjamin | 2022 | 7.1±4.6 | 9.1±3.7 |
|  | Bras | 2024 | -7.5±3.7 | -13.5±5.4 |
|  | Hopman | 2023 | -6.49±2.6 | -7.95±2.21 |
|  | Li | 2022 | 2.99±2.22 | 10.61±2.43 |
|  | Nielsen | 2022 | 12.22±5.14 | 12.22±5.13 |
|  | Song | 2025 | 2.54±2.2 | 7.61±6.01 |
|  | Soysal | 2024 | -8.5±4.5 | -12.9±5.6 |
|  | Zhang | 2025 | -6.4±2.4 | -7.93±1.04 |
| **Post-treatment LASct** | **Author** | **Year** | **Recurrence** | **No recurrence** |
|  | Benjamin | 2022 | 6.2±2.9 | 7.3±1.7 |
|  | Khan | 2023 | 6.6±2.5 | 8.5±3.3 |
|  | Soysal | 2024 | -9.7±7.3 | -15.5±6.3 |
|  | Walek | 2020 | 3.05±2.48 | 5.39±3.5 |
|  | Zhang | 2025 | -6.6±2.6 | -8.93±1.04 |
| **Pre-treatment PALS** | **Author** | **Year** | **Recurrence** | **No recurrence** |
|  | Antonio Moreno-Ruiz | 2019 | 7.55±2.37 | 20.95±8.15 |
|  | Bai | 2018 | 13.03±7.06 | 22.32±11.16 |
|  | Marchandise | 2022 | NR | NR |
|  | Mochizuki | 2017 | NR | NR |
|  | Motoc | 2021 | 20±12.1 | 26.3±10.8 |
|  | Song | 2025 | 6.98±4.17 | 14.7±8.57 |
|  | Zheng | 2023 | NR | NR |

**Supplementary Table 3.** Quality evaluation of included studies using the Newcastle–Ottawa scale

| **Study** | **Selection** | | | | **Comparability** | **Outcome** | | | **Quality scores** |
| --- | --- | --- | --- | --- | --- | --- | --- | --- | --- |
|  | Representativeness of the exposed cohort | Selection of the nonexposed cohort | Ascertainment of exposure | Demonstration that outcome of interest was not present at start of study | Comparability of cohorts on the basis of the design or analysis | Assessment of outcome | Was follow-up long enough for outcomes to occur | Adequacy of follow up of cohorts |  |
| Anastasio2025 | **⭐** | **⭐** | **⭐** | **⭐** | **⭐** | **⭐** | **⭐** | **⭐** | 8 |
| Antonio Moreno-Ruiz2019 | **⭐** | **⭐** | **⭐** | **⭐** | **⭐⭐** | **⭐** | **⭐** |  | 8 |
| Baalman2022 | **⭐** | **⭐** | **⭐** | **⭐** | **⭐⭐** | **⭐** | **⭐** | **⭐** | 9 |
| Bai2018 | **⭐** | **⭐** | **⭐** | **⭐** | **⭐** | **⭐** | **⭐** |  | 7 |
| Benjamin2022 | **⭐** | **⭐** | **⭐** | **⭐** | **⭐⭐** | **⭐** |  | **⭐** | 8 |
| Bras2024 | **⭐** | **⭐** | **⭐** | **⭐** | **⭐⭐** | **⭐** | **⭐** | **⭐** | 9 |
| Cho2023 | **⭐** | **⭐** | **⭐** | **⭐** | **⭐** | **⭐** | **⭐** | **⭐** | 8 |
| Gastl2022 | **⭐** | **⭐** | **⭐** |  | **⭐** | **⭐** | **⭐** |  | 6 |
| Hanaki2020 | **⭐** | **⭐** | **⭐** |  | **⭐** | **⭐** | **⭐** |  | 6 |
| Hao2024 | **⭐** | **⭐** | **⭐** |  | **⭐** | **⭐** | **⭐** | **⭐** | 7 |
| Hong2013 | **⭐** | **⭐** | **⭐** | **⭐** | **⭐** | **⭐** |  | **⭐** | 7 |
| Hopman2023 | **⭐** | **⭐** | **⭐** |  | **⭐** | **⭐** | **⭐** |  | 6 |
| Khan2023 | **⭐** | **⭐** | **⭐** | **⭐** | **⭐** | **⭐** | **⭐** | **⭐** | 8 |
| Li2022 | **⭐** | **⭐** | **⭐** | **⭐** | **⭐⭐** | **⭐** | **⭐** |  | 8 |
| Kiliszek2023 | **⭐** | **⭐** | **⭐** | **⭐** | **⭐** | **⭐** | **⭐** |  | 7 |
| Marchandise2022 | **⭐** | **⭐** | **⭐** | **⭐** | **⭐⭐** | **⭐** | **⭐** |  | 8 |
| Mochizuki2017 | **⭐** | **⭐** | **⭐** | **⭐** | **⭐** | **⭐** |  |  | 6 |
| Motoc2021 | **⭐** | **⭐** | **⭐** | **⭐** | **⭐⭐** | **⭐** | **⭐** |  | 8 |
| Nielsen2022 | **⭐** | **⭐** | **⭐** |  | **⭐⭐** | **⭐** | **⭐** |  | 7 |
| Song2025 | **⭐** | **⭐** | **⭐** | **⭐** | **⭐⭐** | **⭐** | **⭐** |  | 8 |
| Soysal2024 | **⭐** | **⭐** | **⭐** | **⭐** | **⭐⭐** | **⭐** | **⭐** |  | 8 |
| Walek2020 | **⭐** | **⭐** | **⭐** | **⭐** | **⭐⭐** | **⭐** | **⭐** |  | 8 |
| Zeng2024 | **⭐** | **⭐** | **⭐** | **⭐** | **⭐** | **⭐** | **⭐** | **⭐** | 8 |
| Zhang2025 | **⭐** | **⭐** | **⭐** | **⭐** | **⭐⭐** | **⭐** | **⭐** | **⭐** | 9 |
| Zheng2023 | **⭐** | **⭐** | **⭐** | **⭐** | **⭐⭐** | **⭐** |  | **⭐** | 8 |

**Supplementary Table 4.** Meta-regression analysis of association between left atrial strain parameters and AF recurrence

|  | **Coefficient** | **95% CI** | **P** |
| --- | --- | --- | --- |
| **LASr(categorical variable) before treatment** | | | |
| Year | -0.0114579 | -0.0320024to0.0090866 | 0.274 |
| Sample size | -0.0002128 | -0.0007522to0.0003266 | 0.439 |
| Age | -0.0058415 | -0.0311534to0.0194704 | 0.651 |
| Male | 0.0016589 | -0.0056972to0.0090151 | 0.658 |
| BMI | 0.1679307 | -0.0230048to0.3588662 | 0.085 |
| Hypertension | -0.0018205 | -0.0043949to0.0007539 | 0.166 |
| Heart failure | 0.0009052 | -0.003904to0.0057145 | 0.712 |
| Diabetes | 0.0045659 | -0.0073343to0.016466 | 0.452 |
| LVEF | 0.0110736 | 0.0010923to0.0210548 | 0.03 |
| Coronary artery disease | 0.0075825 | -0.0036221to0.0187872 | 0.185 |
| **LASr(continuous variable) before treatment** | | | |
| Year | -0.001064 | -0.0610389to0.058911 | 0.972 |
| Sample size | 0.0000669 | -0.0005501to0.0006838 | 0.832 |
| Age | -0.0042698 | -0.0201494to0.0116097 | 0.598 |
| Male | -0.0005667 | -0.0268485to0.0257151 | 0.966 |
| BMI | 0.0187303 | -0.018946to0.0564067 | 0.33 |
| Hypertension | -0.0008206 | -0.012986to0.0113448 | 0.895 |
| Heart failure | 0.0065139 | -0.0185408to0.0315686 | 0.61 |
| Diabetes | -0.0043173 | -0.0392783to0.0306436 | 0.809 |
| LVEF | -0.0042168 | -0.0227988to0.0143652 | 0.656 |
| Coronary artery disease | -0.001664 | -0.006328to0.0029999 | 0.484 |


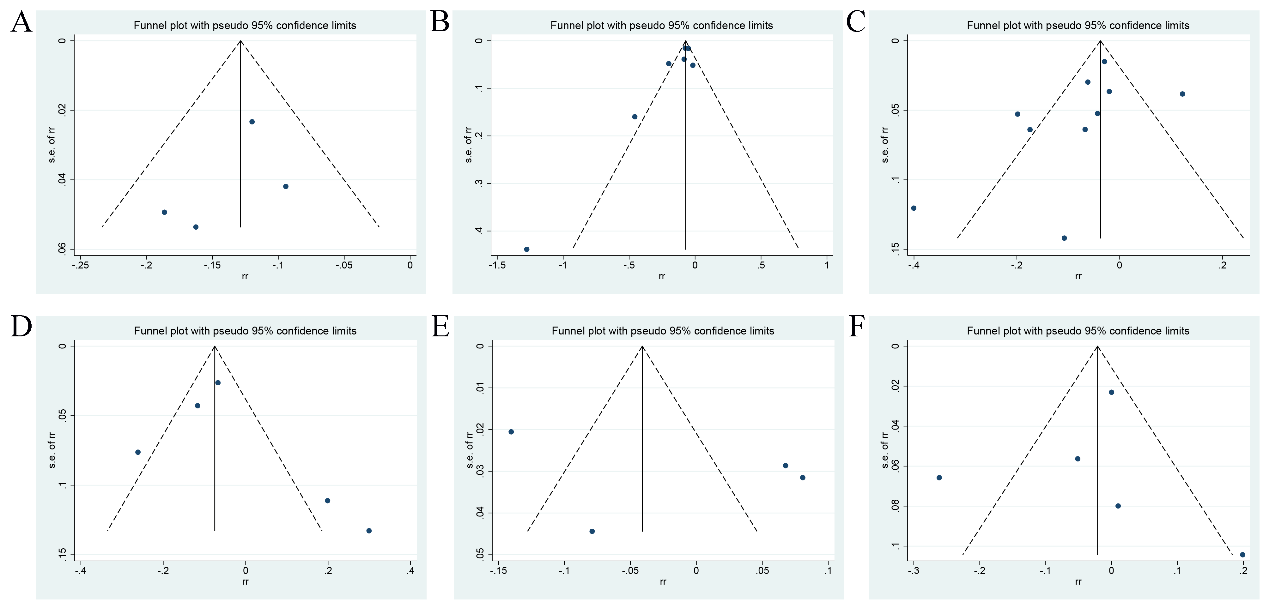


**Supplementary Figure 1.** Funnel plot for association between AF recurrence and PALS before treatment analyzed as a continuous variable (A); for association between AF recurrence and LASr before treatment analyzed as a categorical variable (B) and a continuous variable (C); for association between AF recurrence and LASct before treatment analyzed as a continuous variable (D); for association between AF recurrence and LAScd before treatment analyzed as a categorical variable (E) and a continuous variable (F)


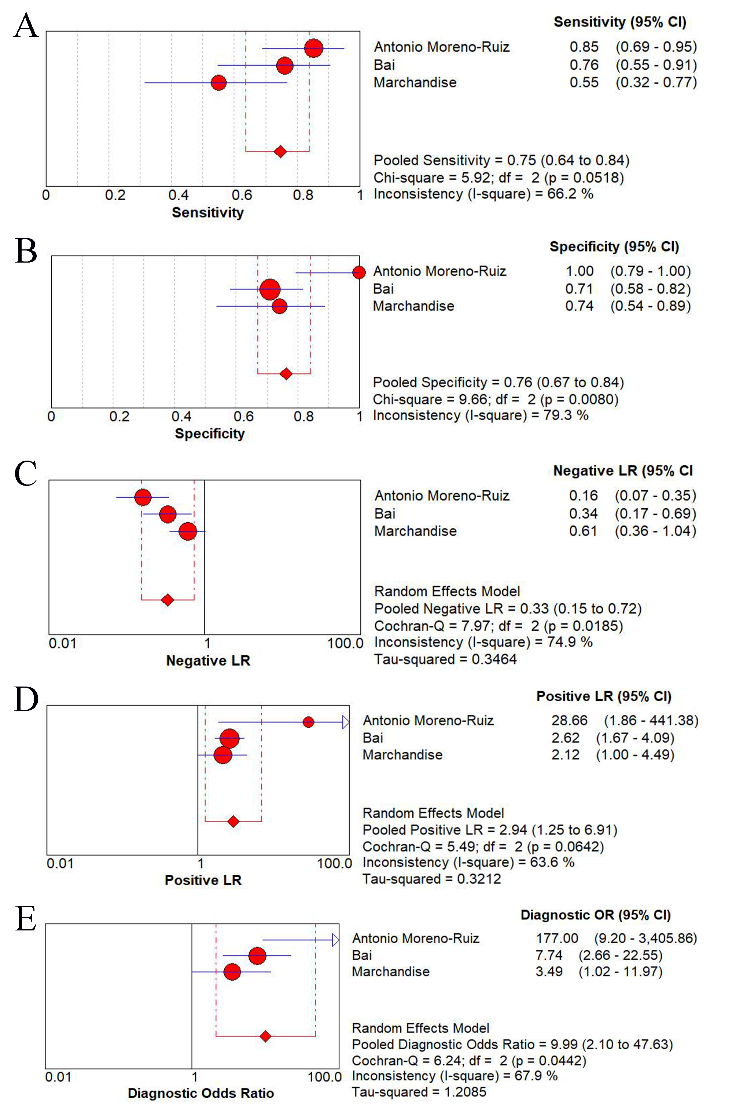


**Supplementary Figure 2.** Pooled sensitivity (A) and specificity (B), NLR (C), PLR (D), and DOR (E) for PALS before treatment


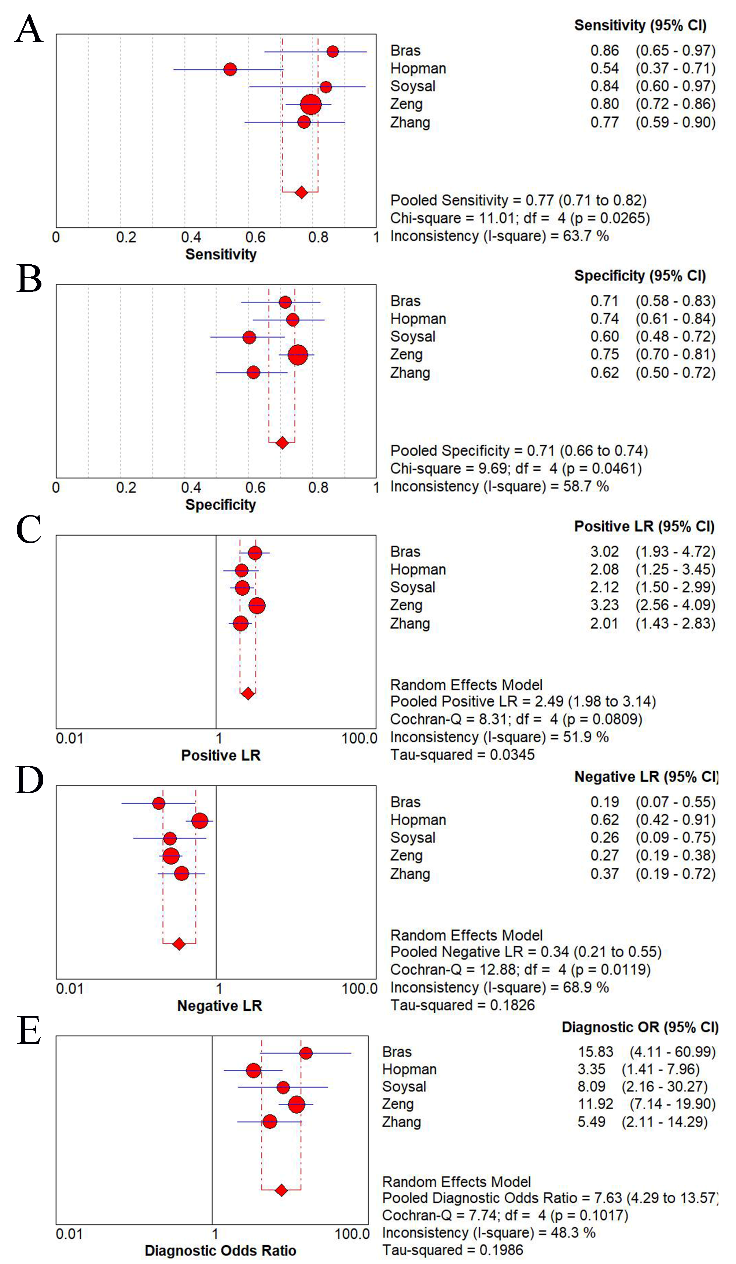


**Supplementary Figure 3.** Pooled sensitivity (A) and specificity (B), NLR (C), PLR (D), and DOR (E) for LASr before treatment
